# Effect of strengths-based, tiered, accessible resources and supports (STARS) for Kids project, a codesigned hybrid service delivery framework to identify and support child development, parent mental health and psychosocial needs among parents and carers of children aged 6 months to 3 years: a randomised controlled trial protocol

**DOI:** 10.1136/bmjopen-2026-122902

**Published:** 2026-09-29

**Authors:** James Rufus John, Jane Kohlhoff, Sini Lambiase, Sophie Nicholls, Maite Diez, Kerrie E Doyle, Karlen Barr, Katherine Boydell, Clare Brennan, Toni Carson, Christa Lam-Cassettari, Jo Cole, Ann Dadich, Joseph Descallar, Amy Finlay-Jones, Melissa Foster, Sharon Goldfeld, Rebekah Grace, Michael Hodgins, Suzy Honisett, Bree Katsamangos, Ilan Katz, Lynn Kemp, Feroza Khan, Luana Kliendienst, Kenny Lawson, Raghu Lingam, Tom McClean, Susan M B Morton, Grainne O’Loughlin, Katarina Ostojic, Andrew Page, Susan Prescott, Shanti Raman, Virginia Schmied, Adam K Walker, Amy Wallace, Rebecca Young, Desiree Silva, Jenny Downs, Susan Woolfenden, Valsamma Eapen, Ngarra Wakulda Aboriginal Community Consultation Group, Aunty Isabell Bungie, STARS for Kids Research Group, Farah Akram

**Affiliations:** 1 School of Clinical Medicine, Faculty of Medicine and Health, UNSW Sydney, Sydney, New South Wales, Australia; 2 Ingham Institute for Applied Medical Research, Liverpool, New South Wales, Australia; 3 Academic Unit of Infant, Child, and Adolescent Psychiatry Services, South Western Sydney Local Health District, Liverpool, New South Wales, Australia; 4 Karitane, Villawood, New South Wales, Australia; 5 The Kids Research Institute Australia, Nedlands, Western Australia, Australia; 6 Faculty of Medicine and Health, The University of Sydney, Sydney, New South Wales, Australia; 7 School of Rural Medicine, Charles Sturt University, Orange, New South Wales, Australia; 8 Black Dog Institute, Randwick, New South Wales, Australia; 9 First Steps Count, Taree, New South Wales, Australia; 10 School of Business, Western Sydney University, Penrith, New South Wales, Australia; 11 School of Population Health, Curtin University, Perth, Western Australia, Australia; 12 Biripi Aboriginal Corporation Medical Centre, Purfleet, New South Wales, Australia; 13 Murdoch Children’s Research Institute, Parkville, Victoria, Australia; 14 Department of Paediatrics, The University of Melbourne, Melbourne, Victoria, Australia; 15 Transforming Early Education and Child Health (TeEACH) Strategic Research Initiative, Western Sydney University, Campbelltown, New South Wales, Australia; 16 Mission Australia, Sydney, New South Wales, Australia; 17 Social Policy Research Centre, Faculty of Arts and Social Science, University of New South Wales, Sydney, New South Wales, Australia; 18 School of Nursing and Midwifery, Western Sydney University, Penrith, New South Wales, Australia; 19 Translational Health Research Institute, Western Sydney University, Penrith, New South Wales, Australia; 20 Uniting NSW.ACT, Kingston, Australian Capital Territory, Australia; 21 Research Institute for Innovative Solutions for Wellbeing and Health, University of Technology Sydney, Sydney, New South Wales, Australia; 22 School of Medicine, The University of Western Australia, Perth, Western Australia, Australia; 23 Community Paediatrics, South Western Sydney Local Health District, Liverpool, New South Wales, Australia; 24 Neuroscience Research Australia, Randwick, New South Wales, Australia; 25 Joondalup Health Campus, Joondalup, Western Australia, Australia; 26 Institute for Women, Children and their Families, SLHD, Camperdown, New South Wales, Australia

**Keywords:** Community child health, Delivery of Health Care, Integrated, Social Support, Randomized Controlled Trial, MENTAL HEALTH, Developmental neurology & neurodisability

## Abstract

**Introduction:**

Inequitable access to early developmental support persists across both universal and specialised services for priority populations. However, real-world evidence on the impact of implementing proportionate universalism framework (universal plus targeted supports commensurate with needs) within service systems is limited. To address this gap, we aim to implement and evaluate a codesigned model of care called the Strengths-based, Tiered, Accessible Resources and Supports for Kids (hereafter, STARS for Kids) model to improve early identification and delivery of appropriate support for child developmental vulnerabilities, parental mental health and family psychosocial needs across the first 2000 days of life, from pregnancy to the start of school.

**Methods and analysis:**

This study will be conducted in three phases alongside the codesign phase that will be integrated across the life of the study. First, the impact evaluation phase will be conducted using a multisite, individually randomised controlled trial to evaluate the effectiveness of the STARS for Kids tiered care model (intervention group) against enhanced usual care (control group). Parents/carers of children aged 6 months to 3 years (140 per site) will be recruited using opportunistic contacts in three sites: (i) a multicultural site with a high immigrant population in South Western Sydney in New South Wales (NSW), Australia; (ii) a regional/rural site including an Aboriginal community on Biripi land in Taree in NSW; and (iii) a low socioeconomic site, the City of Wanneroo in Western Australia. Additionally, a nested trial will evaluate the My Toddler and Me Enhanced Video Programme intervention against enhanced usual care among children identified with emotional dysregulation. Second, the implementation evaluation phase will be conducted with consumers and service providers (approximately 15–20 per group and site). Underpinned by the consolidated framework for implementation research, it will evaluate the perceived feasibility, satisfaction, benefits, barriers and facilitators of the model. Third, the economic analysis and dynamic simulation modelling phase will ascertain the cost–benefits and model the impact of the STARS for Kids intervention across the life course for children and families. The primary outcome (parent or carer-reported completion of child developmental checks) and secondary outcomes (changes in child developmental vulnerabilities, parental mental health and family psychosocial needs between the intervention and control groups) will be compared by intention-to-treat analysis using generalised mixed models to account for group, time and interactions. Interview data from the implementation evaluation phase will be thematically analysed to identify, refine and integrate themes representing participant experiences.

**Ethics and dissemination:**

The South Western Sydney Local Health District Human Research Ethics Committee approved this protocol for the South Western Sydney and Taree sites in NSW (2024/ETH01146) and Ramsay Ethics approved this protocol for the Wanneroo site in Western Australia (2025/ETH/0002). Site-specific research governance approvals were granted for all three sites. Given the substantial proportion of Aboriginal and/or Torres Strait Islander communities in Taree, an Aboriginal Health and Medical Research Council ethics application was approved for the Taree site (2478/25). Findings from this study will be published in reports and refereed journals and presented at appropriate conferences and community forums following consultations with the relevant steering committees and consumer groups.

**Trial registration number:**

ACTRN12624000806561.

**Strengths and limitations of this study**

- The Strengths-based, Tiered Accessible Resources and Supports for Kids intervention harnesses opportunistic contacts with health, early childhood, education and social services to facilitate systematic identification and referral of children and families according to their needs.
- The trial and intervention procedures were codesigned with families from Aboriginal and culturally and linguistically diverse communities, incorporating culturally responsive and strengths-based approaches throughout the study design and implementation.
- The study will integrate a randomised controlled trial, implementation science, economic and dynamic simulation modelling to comprehensively assess effectiveness, implementation, costs and long-term outcomes.
- The study may be limited by parent/carer self-report of the primary outcome, differential engagement and contamination between study arms, attrition among priority populations and limited power in the nested trial.
- Given this study focuses on priority population groups at three specific sites, the generalisability of the model across other settings is uncertain.

## Background

### Burden of developmental vulnerability among priority populations

 Supporting the early years of a child’s life is a national priority to ensure all children in Australia thrive as per the National Mental Health Strategy^1^ and the National Action Plan for the health of children and young people: 2020–2030.^2^ However, the 2024 Australian Early Development Census, a nationwide data collection of early childhood development, shows that child developmental vulnerability continues to rise, with one in four children starting school developmentally vulnerable - the highest since the census started in 2009.^3^ These rates are two times as high among children from priority populations, such as children from culturally and linguistically diverse (CALD), Aboriginal and Torres Strait Islander, regional/rural and socio-economically disadvantaged communities. These populations continue to face financial, structural and systemic barriers to access health and social services.^3 4^ Early developmental vulnerability imposes a triple burden on children and families across health (eg, poorer physical and mental health and higher rates of chronic disease),^4 5^ psychosocial (eg, reduced school readiness, social participation and increased family stress)^6 7^ and economic domains (eg, higher long-term service use, lower educational attainment and workforce participation and greater intergenerational disadvantage).^8 9^ These inequities are compounded by the complex, poorly coordinated and inefficient nature of the Australian child and family service system with fragmentation, duplication and service delivery gaps.^10 11^ Although early identification and support during the first 2000 days of life yield the best outcomes and return on investment,^12–14^ developmental checks remain low and accessibility to these checks is inequitable.^15 16^ Further, there is an inverse care law, whereby children from priority populations with the highest developmental risk are least likely to access services early.^17^ This reflects a legacy of historical policies and practices that have resulted in a failure to build safe, trusting relationships with people in these populations in healthcare settings.^18^ Additionally, despite the implementation of state and federal programmes, uptake continues to be low, and there are no systematic mechanisms to engage families early so that developmental differences can be identified when they emerge. Without timely intervention, these children face systemic barriers that may put them at risk of behind before they even step into a classroom.

### Shift towards strength-based and integrated approaches

To date, much of the research about the first 2000 days, from pregnancy to start of school, focuses on identifying risk and reducing adverse outcomes. Further, current approaches to identifying and supporting children with developmental needs fail to consider the broader social determinants and the impact of historical discriminatory policies, such as parental mental health, family psychosocial needs, unmet legal need and other structural inequities.^19–21^ In particular, racism and systemic racism operate as fundamental social determinants of health outcomes, with intergenerational effects that influence access and engagement with resources and supports, and trust in health and social care systems among priority populations.^18^ In practice, children frequently present with multiple co-occurring symptoms arising from overlapping pathways that are poorly captured by risk-focused models. Such approaches limit preventative care by placing excessive emphasis on diagnoses rather than responding to early developmental concerns and the broader socio-economic contexts in which children and families live, which are central to delivering patient-centred and prevention-focused care. This in turn misses opportunities for holistic care.^22^ Conversely, there is a growing advocacy for a shift towards strengths-based, holistic and integrated approaches informed by ecological frameworks (eg, Bronfenbrenner^23^), which recognise the dynamic interplay between the child, family, community and broader systems. Such approaches emphasise early support, family strengths and integrated service responses to better engage parents, strengthen families and communities and improve long-term developmental outcomes.^24^

### Current evidence on integrated tiered models of care for children and families

Consistent with proportionate universalism,^25^ integrated tiered models of care provide a structured framework to deliver tailored support based on a family’s levels of risk and needs, ranging from universal services to targeted and intensive interventions for those experiencing greater vulnerability.^26^ Integrated tiered approaches can improve access, efficiency and equity by tailoring interventions based on needs and reducing fragmentation across care pathways.^27 28^ Integrated tiered approaches have been implemented digitally or in-person. For instance, digital innovations can aid detection and intervention for developmental vulnerabilities via a more flexible and efficient developmental surveillance,^29 30^ whereas in-person service navigation approaches can improve access to health services among parents or carers of infants and young children.^31–34^ Additionally, there is some international evidence on place-based programmes, such as the UK’s Sure Start programme,^34^ leading to long-term social, emotional and behavioural developmental outcomes. However, no national or international models integrate the early identification of needs alongside coordinated, integrated tiered care pathways addressing child development, parental mental health and unmet social care needs within a unified model.

Evidence for models of care incorporating codesign and cultural adaptation is particularly sparse. Most evaluated models have been developed and tested in high-income, predominantly Anglo-centric contexts, with poor transferability to Aboriginal and Torres Strait Islander communities, refugee and migrant families or rural and remote populations.^35–37^ Although implementation science highlights tensions between fidelity and contextual adaptation in real-world settings, few studies have prospectively examined how integrated tiered models perform across diverse settings or used community codesign to inform their development.^38^ Further, families’ needs in the early years are heterogeneous, dynamic and shaped by interacting psychosocial and structural factors.^39^ These needs often fluctuate over time and might not align neatly with eligibility thresholds for targeted services, resulting in missed opportunities for early support. A one-size-fits-all model is a continuation of ‘business as usual’ approach that provides insufficient support for families with greater or more complex needs, while targeted approaches risk families with emerging or less visible vulnerabilities falling through the cracks.^40^ As such, there is an urgent need for codesigned models that can flexibly provide support in response to changing needs, consistent with proportionate universalism, to ensure timely, equitable and sustained engagement with services across diverse populations.

### Study aims and phases

This study aims to implement and evaluate the codesigned Strengths-based, Tiered, Accessible Resources and Supports for Kids (hereafter, STARS for Kids) model of care to systematically improve the screening and support for child developmental checks, parental mental health and family psychosocial needs. This model of care comprises three phases: (1) an impact evaluation phase using a multisite randomised controlled trial (RCT) to evaluate the effectiveness of the STARS for Kids tiered care model on parent or carer uptake of developmental checks, child developmental concerns, parental mental health and family psychosocial outcomes; (2) an implementation evaluation phase to ascertain parent, carer and stakeholder (eg, service providers and policymakers) experiences with the STARS for Kids intervention; and (3) an economic analysis and dynamic simulation modelling phase to evaluate the cost and benefits of the STARS for Kids intervention, including both within-trial impacts and modelling of longer-term outcomes.

This protocol has been prepared in accordance with the Standard Protocol Items: Recommendations for Interventional Trials (SPIRIT guidelines) statement and checklist (online supplemental file 1).^41^

## Methods and analysis

### Study design

In the impact evaluation phase (phase I), a multisite parallel group RCT will be conducted with two components: a main trial and a nested trial.

The main trial will involve randomising participants to receive either the STARS for Kids tiered care model (intervention group) or enhanced usual care (control group). This component evaluates the effectiveness of the overall tiered model of care across the full study population, including outcomes related to parent or carer uptake of developmental checks, child developmental concerns, parental mental health and family psychosocial needs.

The nested RCT will be conducted within the main trial among a subsample of children identified as having emotional dysregulation (ie, poor ability to manage emotional responses), based on the Survey of Well-being of Young Children (SWYC) scores (Baby Paediatric Symptom checklist score ≥3 on any of the three subscales or Preschool Paediatric Symptom checklist score ≥9). Randomisation will be maintained from the main trial, such that participants remain in their originally allocated study arm, thereby minimising contamination between intervention groups. Within this clinically defined subgroup requiring higher-intensity support, the nested trial will evaluate the effectiveness of a specific tier 3 intervention, namely, the My Toddler and Me Enhanced Video Program (MTAM-EVP) compared with enhanced usual care (control group) (figure 1).

**Figure 1:**
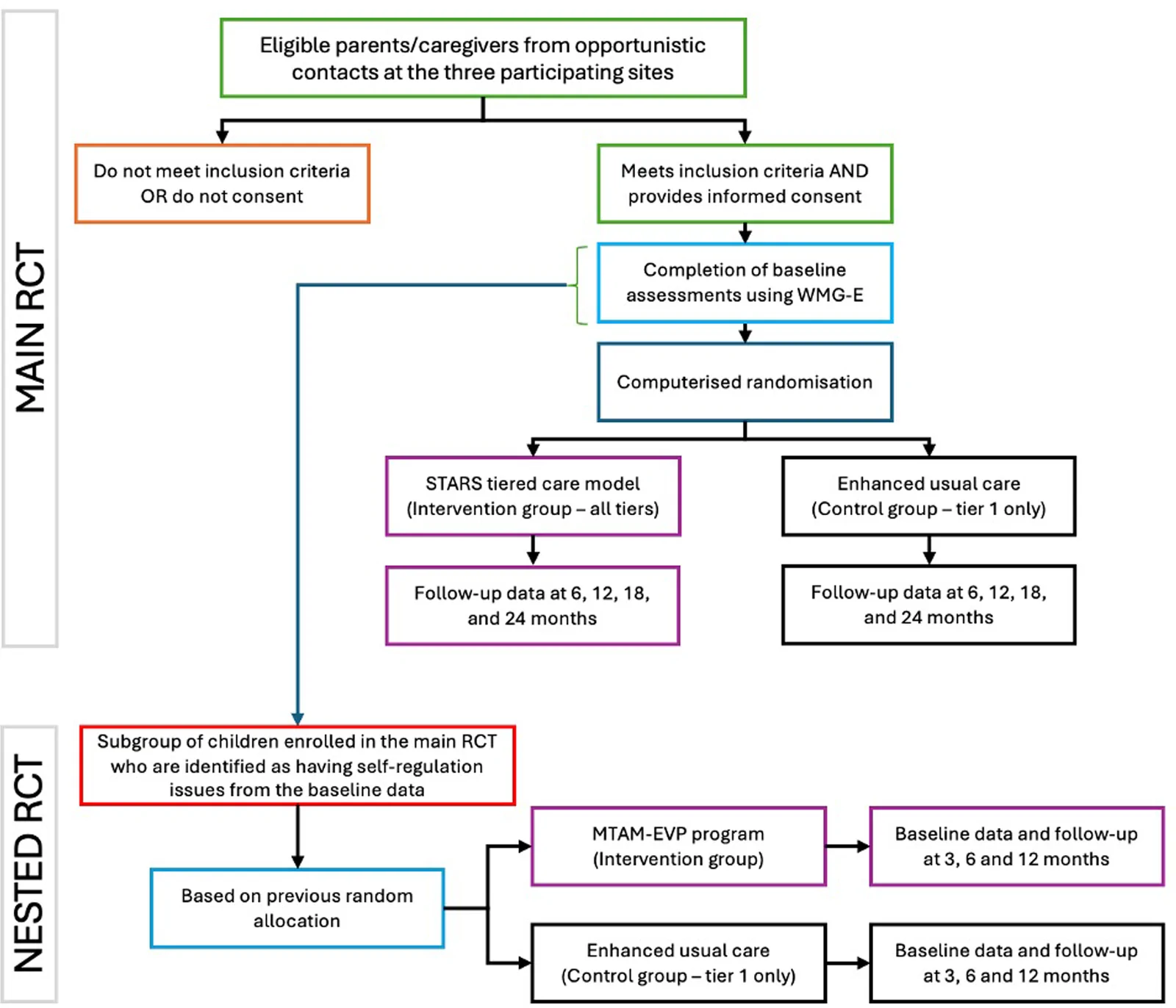
Study flowchart. MTAM-EVP, My Toddler and Me Enhanced Video Program; RCT, randomised controlled trial; STARS for Kids, Strengths-based, Tiered, Accessible Resources and Supports for Kids; WMG-E, Watch Me Grow-Electronic.

For the implementation evaluation phase (phase II), the consolidated framework for implementation research (CFIR)^42^ and Proctor and colleagues’ implementation metrics^43^ will be used. Interviews will be conducted with a random subset of parents and carers who participated in the trial phase to ascertain their experiences with the STARS for Kids intervention. The interviews will address perceived feasibility, satisfaction and benefits as well as barriers, including suggestions to improve the implementation of the intervention.

For the economic analysis and dynamic simulation phase (phase III), the primary and secondary outcomes measures will be used to model the impact of the STARS for Kids intervention across the life course for children and families.

### Study sites

The three sites include Liverpool/Fairfield and Bankstown in South Western Sydney (SWS), New South Wales (NSW), given these sites are home to culturally and linguistically diverse (CALD) communities; Taree in NSW (Biripi Country) due to its substantial Aboriginal and Torres Strait Islander population and its rural/regional context; and the City of Wanneroo in Western Australia (WA) given this site is home to a community of limited means.

### Study participants

#### Parents or carers

The inclusion criteria are as follows: (a) parents or carers (aged ≥15 years) of children (aged 6 months to 3 years); (b) who reside in one of the three sites and attend child and family health services, refugee health services, supported playgroups, preschools and early childhood education centres, parenting groups, non-government organisations, general practices, special paediatric clinics and/or other state and national child and family programmes; and (c) ability to provide informed consent.

The exclusion criteria are as follows: (a) not having a child within the age range in the inclusion criteria, (b) unable to provide informed consent and (c) residence outside of the study sites.

For the nested trial component, a subsample of participants from the main trial will be eligible if their child is between 12 and 36 months old and is identified as experiencing emotional dysregulation based on screening within the STARS for Kids model of care.

#### Service providers

Service providers will be recruited for the implementation evaluation phase.

The inclusion criteria are as follows: (a) service providers who deliver health, social or community services relevant to child and family well-being at one of the study sites, and (b) ability to provide informed consent.

The exclusion criteria are as follows: (a) service providers not involved in service delivery within the participating sites, or (b) inability to provide informed consent.

### Recruitment and consent

For the impact evaluation phase, parents and carers who meet the inclusion criteria will be invited to participate via a flyer/poster with a quick response (QR) code. The flyer/poster alongside other promotional material (eg, short video) will also be advertised via newsletters or email promotion to the appropriate health department staff directories, community centres and as content for local Facebook posts and other appropriate social media platforms. Given the substantial proportion of CALD communities in SWS, flyers will be translated to Chinese (Cantonese and Mandarin), Arabic and Vietnamese. Given the potential for a high proportion of Aboriginal and Torres Strait Islander participants in Taree, the flyers will be codesigned with the Ngarra Wakulda Aboriginal Community Consultation Group in Taree. Additionally, the researchers will facilitate information sessions at relevant services (eg, playgroups and community centres) to explain the study and address queries and concerns.

Parents and carers who are interested in the study will be able to use the QR code to source the eligibility criteria and access the participant information sheet and consent form (PISCF). The PISCF will provide information about the aim and reason for research, its risks and benefits and the voluntary nature of the study. Participants can elect to participate immediately or engage in a conversation with a nominated researcher (via telephone or email) and participate later. The PISCF states that if participants change their minds about joining the study, they can withdraw at any stage without reason or consequence. Additionally, an alternative Easy Read PISCF will be provided as an option for those with limited English proficiency. Translated versions of PISCF or interpretation services for participants will be provided for those who prefer to speak in a language other than English. Further, parents and carers with technological (eg, internet access/issues) or literacy issues will be supported by the service providers the families are engaged with and/or staff who provide the flyer or introduce the parents or carers to the study.

For the implementation evaluation phase, a subsample of parents and carers enrolled in the intervention group of the RCT will be approached for the qualitative subproject using convenience sampling. They will be provided with a PISCF providing information about the aim and reason for qualitative research, its risks and benefits and the voluntary nature of the study and asked to indicate whether they consent to being contacted for an interview (30-60 min). Those who consent to being contacted will be invited to participate in the qualitative interviews at the 12-month and 24-month timepoints. Those who are interested to participate will be provided a detailed PISCF to provide consent via the aforesaid ways.

For both the impact and implementation phases, eligible participants will be able to consent immediately or 1-2 weeks later. Every effort will be made to ensure it is safe for potential participants to autonomously consent. There will be no pressure to participate, and it will be made clear that declining to participate will have no consequence with healthcare provision.

#### Sample size and power calculation

##### Impact evaluation phase

100 children per site (50 per group; total n=300 across three sites) will provide 80% power to detect a 30% absolute increase in uptake of well-child checks (30%–60%) in the intervention group at a two-sided α of 0.05. Allowing for 40% attrition, 140 participants per site will be required (total n=420). For the nested RCT, a subgroup of children enrolled in the main trial who are identified as having self-regulation issues will form the study group; this is expected to be 20% (n~40 children in each site). Using generalised linear mixed models with the random effect of participants and fixed effects (treatment and time), approximately 90 participants are required to test the group × time interaction with a power of 0.80, assuming a small-to-moderate interaction effect (*f*=0.15). This sample is also sufficient to find the 30% improvement in uptake of well-child checks between the two groups at 12 months, assuming an autocorrelation of up to 0.95. The feasible sample of 120 therefore allows for 25% loss to follow-up.

##### Implementation evaluation phase

The sample size of approximately 15–20 families in the intervention group who participated in the study and 15–20 stakeholders (eg, service providers and policymakers) was established using the concept of ‘information power’.^44^

### Randomisation, blinding and allocation concealment

Participants will be randomised to either the intervention or control group via the Watch Me Grow-Electronic (WMG-E) platform, described later. Computerised randomisation software will be used to create a randomisation table via REDCap (a secure web application for building and managing online surveys and databases), ensuring a 1:1 ratio. Blinding of participants and service providers is not feasible because of the nature of the intervention. Allocation concealment will be ensured through centralised, computer-generated randomisation implemented via REDCap by an independent biostatistician. Additionally, statistical analyses will be undertaken by investigators blinded to allocation.

### Intervention

The intervention group will receive the codesigned STARS for Kids tiered care model. Further details about the ongoing codesign process and activities to refine the model are described elsewhere.^45^ The intervention comprises universal and the full tiered care services, detailed as follows.

#### Universal service

The WMG-E platform is a digital front door to systematically identify the child’s developmental, parental mental health and family social care needs. It is effective^46^ and acceptable^30^ to families for the early identification of child developmental, parental mental health and family psychosocial needs. WMG-E contains in-built logic to identify children at developmental risk at the point of care to aid shared decision-making. Results are automatically transmitted to a parent or carer and the service provider who completes further assessments as needed. Once engaged, the platform issues automated reminders to the parent or carer, prompting them to complete the surveys at the next follow-up timepoints (see figure 2).

**Figure 2:**
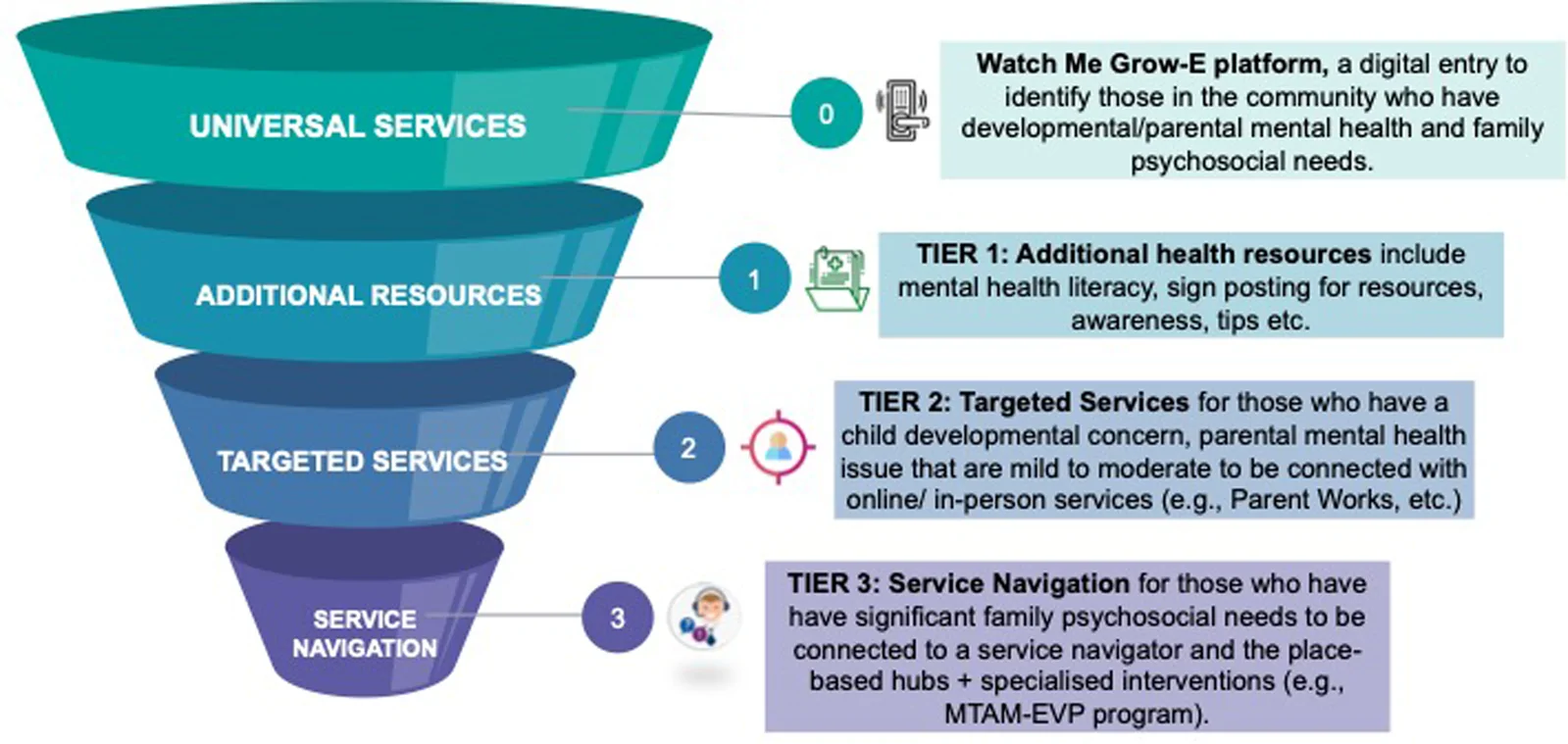
STARS for Kids tiered care model. MTAM-EVP, My Toddler and Me Enhanced Video Program; STARS for Kids, Strengths-based, Tiered, Accessible Resources and Supports for Kids.

#### Tiered service approach

Based on complexity and needs, services are tiered as follows.

##### Additional resources (tier 1)

Parents or carers will be provided with anticipatory guidance through a resource pack that includes health and developmental literacy materials, practical tips and content aimed at building awareness of child development and early signs of concern. The resource pack is codesigned with parents/carers, service providers and other key stakeholders (including the Raising Children Network) and will include information on locally available universal services (eg, child and family health services and community-based supports) to improve awareness and facilitate access.

##### Targeted services (tier 2)

Parents or carers with mild child developmental concerns, mild mental health issues or one or more unmet social care needs will be connected via digital navigation with online and/or in-person services (eg, Triple P online and parenting group interventions) available at each site.

##### Service navigation, place-based and specialised interventions (tier 3)

Parents and carers with complex health and family psychosocial needs will be linked with an in-person service navigator who will connect them with relevant health (eg, developmental assessments and intervention) and social care providers (eg, place-based hubs, MTAM-EVP intervention, intensive parenting interventions, strengths-based approaches to increase family and community connectedness) for wraparound care with a warm handover to referred services and continuity of care to ensure the referral needs are met. In WA site, tier 3 intervention will be delivered digitally by an online service navigator, with interventions for child development and parental mental health needs matched to individual family needs and supported by wraparound social care, including warm handover and ongoing support to facilitate engagement and ensure needs are met.

##### My Toddler and Me Enhanced Video Program

As part of the tier 3 service offering within the STARS for Kids model, the MTAM-EVP will be evaluated through a nested trial. This programme aims to enhance parent/caregiver self-efficacy around emotions and empathy, parent/caregiver use of supportive responses to negative toddler (12–36 months) emotions and self-regulation in toddlers. The programme consists of 4 weekly, 30 min, individual telehealth sessions with a trained clinician, and a fifth follow-up session after a 2–3 week break. In each of the four sessions, the parent/caregiver views a video from the ‘My Toddler and Me’ video series (one video per session) and then engages in discussion with the clinician about ways to implement the new parenting strategies presented. The fifth session provides an opportunity for the parent/caregiver to ask any last questions and reflect on the material presented in the programme as a whole. The video series draws on the principles of Parent-Child Interaction Therapy – Toddler^47^ and was designed to normalise the challenges of toddlerhood and provide practical strategies to support children’s emotional well-being and development. Video topics include using positive parenting to foster play, development and secure parent–child relationships; supporting toddler emotional regulation; enhancing parental emotion regulation and self-care; and promoting healthy and positive toddler exploration. The videos were co-designed with English-speaking, Arabic-speaking and Vietnamese-speaking parents and health professionals to ensure cultural relevance, sensitivity and accessibility. Each video is approximately 3 min long, features simple and clear messaging and uses animation with multilingual voice-over narration.

### Control group

Parents or carers in the control group will receive enhanced usual care, comprising tier 1 additional resources as described above at baseline. Enhanced usual care was selected as the comparator to reflect current real-world service provision while ensuring all participants receive a minimum level of support. This approach is ethically appropriate for a community-based population and enables evaluation of the effectiveness of the STARS for Kids tiered model over and above usual care.

No concomitant care will be prohibited for either group, as the trial is designed to reflect real-world service delivery.

### Data collection

#### Demographic information

Child age and sex, as well as parent age, sex, ethnicity including if they identify as Aboriginal and/or Torres Strait Islander (at Taree site), and country of birth, will be self-reported only at baseline in both groups.

### Primary outcome measures

#### Developmental checks

Parent and carer reports of completed developmental checks from the NSW Blue Book or WA Purple Book will be determined at baseline (ie, checks completed before study participation). The Blue and Purple Book programmes recommend that health and developmental checks are completed at 6, 12, 18, 24, 36, 48 and 60 months. Baseline reporting will capture whether families have engaged with child and family health services for recommended checks up to the child’s age at enrolment. Follow-up assessments will occur at 6, 12, 18 and 24 months after baseline (ie, study follow-up timepoints and not child age milestones) and will assess ongoing engagement with recommended developmental checks over the study period in both groups. In the intervention group, families will also receive prompts via the WMG-E platform to complete developmental checks at the follow-up timepoints, whereas the control group will not receive these prompts. Comparisons between groups will assess the effectiveness of the intervention in supporting sustained engagement with recommended developmental checks over time.

### Secondary outcome measures

#### Developmental milestones

Learn The Signs Act Early (LTSAE)^48^ will be used, which includes ‘red flag’ questions with ‘yes’, ‘no’ and ‘not sure’ response categories, for children aged 2 months to 5 years. LTSAE is used to monitor children’s social, emotional, language, cognitive and physical development. The WMG-E has a built-in algorithm that determines which LTSAE is done at what age based on the midpoint (ie, if a child is <9 months old, parents and carers will be requested to complete the 6-month LTSAE, whereas if the child is >9 months old, a 12-month LTSAE will be completed). The Quantitative Checklist for Autism in Toddlers-10 (Q-CHAT-10)^49^ will also be used to identify autism traits among children aged 16–35 months. Q-CHAT-10 includes 10 questions, with each question asking about a specific behaviour, and parents and carers respond based on how frequently their child displays it.

If a concern is raised on the aforesaid screening questionnaires, a secondary screener, SWYC^50^ will be used. This is a comprehensive developmental and behavioural screening tool for children from birth to 5 years old. It assesses communication, gross motor skills, fine motor skills, problem-solving, personal–social skills and emotional/behavioural health. Given that several of the developmental milestones will be captured using LTSAE, the SWYC’s baby and preschool Paediatric Symptom Checklist (PSC) will be used as a secondary screener to minimise research fatigue.

#### Parent and carer mental health

The Kessler Psychological Distress Scale-10 (K10) questionnaire will capture parent and carer distress.^51^ K10 assesses psychological distress over the past 4 weeks by asking how often the responder felt nervous or hopeless on a 5-point scale: all the time, most of the time, some of the time, a little of the time and none of the time.

#### Unmet psychosocial needs

A modified WE CARE questionnaire^52^ will be used, consisting of six (yes or no) items querying childcare, employment, homelessness, food security, education and utilities. If parents and carers answer ‘yes’ to a question, they are asked whether they would like support in the nominated area (yes, no or maybe later). Questions regarding experiences of racism will be included, due to the relation between racism and health access barriers, inequities and unmet social needs.

All the aforesaid assessments will be collected at baseline and at 6, 12, 18 and 24 months in the intervention group, whereas the control group will complete these assessments at baseline and at 12 and 24 months to minimise research fatigue.

#### Health service use

Self-reported data on families’ service use will be collected at baseline and at 6, 12, 18 and 24 months from the intervention group and at baseline and at 12 and 24 months from the control group via the WMG-E platform. For both groups, service providers will also be asked to complete a data log to record the number of children seen or discussed, the number of healthcare providers attending case discussions, the number of emails and telephone calls fielded and the waiting time to access the services.

To minimise research fatigue in the control group, a brief semi-structured questionnaire will be administered at 6 and 18 months. This will ask participants whether they have sought help for any needs identified at baseline and whether they encountered barriers, rather than requesting them to complete the full set of questionnaires again.

#### Service satisfaction

A patient satisfaction questionnaire will be used to ascertain overall satisfaction in both groups, whereas the Navigation Satisfaction Tool (NAVSAT)^53^ will be used to ascertain service navigation satisfaction within the intervention group. Data will be self-reported at 12 and 24 months via the WMG-E platform.

#### Economic modelling

The EuroQol five-dimension questionnaire five-levels (EQ-5D-5L) is a standardised generic instrument to measure (preference-weighted) health-related quality of life. It consists of five dimensions: mobility, self-care, usual activities, pain/discomfort and anxiety/depression, each rated on five levels of severity. It also includes a Visual Analogue Scale (VAS) for overall health assessment. The EQ-5D-5L is widely used in clinical and economic evaluations to assess health outcomes and inform healthcare decision-making. Additionally, the Caregiver Indirect and Informal Care Cost Assessment Questionnaire (CIIQ) quantifies the economic and personal burden of caregiving for use in health economic studies and policy development. It evaluates factors such as time spent on caregiving, productivity loss, out-of-pocket expenses and the impact on the carer’s quality of life.

### Nested trial measures

Eligible and consented parents/carers of children in the nested trial will complete the below measures.

*Patient-Reported Outcome Measurement Information System Early Childhood Well-being Measure* (Flexibility and Frustration Tolerance subscales) will assess toddler self-regulation.^54^

*Multidimensional Assessment Profile of Disruptive Behaviour (Infant-Toddler) – Temper Loss* will measure the frequency and intensity of early irritability and tantrum behaviours, distinguishing normative misbehaviour from clinically significant patterns.^55^

*Tool to Measure Parenting Self-Efficacy (TOPSE)* will assess carers’ self-efficacy around showing affection, empathy and playing/enjoying their child. To reduce participant burden and assess intended programme outcomes, only three of the eight TOPSE subscales will be collected: emotion and affection; empathy and understanding; and play and enjoyment.^48^

*Coping with Toddlers Negative Emotions Scale (CTNES)* will assess parent and carer responses to children’s negative emotions, identifying coping strategies that promote or hinder children’s emotional regulation and social competence.^56^ To reduce participant burden, a revised form of the CTNES will be used comprising 9 of the 12 scenarios.

The aforesaid nested trial assessments will be collected at baseline and at 3, 6 and 12 months from the intervention and control groups.

A summary of the measures collected at each timepoint is presented table 1.

**Table 1:** Study schedule of data collection

Measures	Baseline	Nested trial only (~3 months)	6-month follow-up	12-month follow-up	18-month follow-up	24-month follow-up
Sociodemographic information	✔					
Parent-reported completion of child developmental checks	✔		✔	✔	✔	✔
Service use	✔		✔	✔	✔	✔
Developmental surveillance, Learn The Signs Act Early	✔		✔	✔	✔	✔
Autism screening, Quantitative Checklist for Autism in Toddlers–10	✔		✔	✔	✔	✔
Survey of Well-being of Young Children - Baby and Preschool Paediatric Symptom Checklist	✔		✔	✔	✔	✔
Parental mental health (Kessler Psychological Distress Scale-10)	✔		✔	✔	✔	✔
Unmet psychosocial needs (WE CARE)	✔		✔	✔	✔	✔
Questions about racism	✔		✔	✔	✔	✔
EuroQol five-dimension questionnaire five-levels	✔		✔	✔	✔	✔
Caregiver Indirect and Informal Care Cost Assessment Questionnaire	✔		✔	✔	✔	✔
Participant satisfaction questionnaire*			✔	✔	✔	✔
Navigation Satisfaction Tool						✔
Qualitative interviews				✔		✔
Nested trial						
Patient-Reported Outcome Measurement Information System Early Childhood Well-being Measure†	✔	✔	✔	✔		
Multidimensional Assessment Profile of Disruptive Behaviour (Infant-Toddler) – Temper Loss†	✔	✔	✔	✔		
Tool to Measure Parenting Self-Efficacy – three subscales only: (1) emotion and affection subscale, (2) empathy and understanding subscale; and (3) play and enjoyment subscale†	✔	✔	✔	✔		
Coping with Toddlers Negative Emotions Scale†	✔	✔	✔	✔		

* Intervention group only.

† Nested randomised controlled trial participants only.

WE CARE, Well-Being and Caregiver Assessment and Research Evaluation.

#### Qualitative interviews

To explore factors influencing implementation, interviews will be guided by the CFIR^42^ and Proctor and colleagues’ implementation metrics^43^ (table 2), which provides comprehensive coverage of multi-level implementation determinants and has evidence-based utility in qualitative health research. The CFIR constructs will inform both the interview guide design and subsequent framework analysis, with inductive coding applied alongside deductive coding to capture any contextually relevant factors not represented within the framework. Data will be collected at 12 and 24 months from both groups.

**Table 2:** Implementation evaluation metrics

Measure	Questions addressed by each implementation measure
Acceptability	Do consumers and service providers perceive the service as agreeable?
Adoption	To what extent do consumers and service providers perceive the service will be adopted in routine practice?
Appropriateness	Do stakeholders perceive the service as relevant and useful?
Fidelity	Is the service applied as intended?
Coverage	How many service users who were approached agreed to complete the service?
Cost	Are there any additional costs for services to implement the service?
Sustainability	What are the factors that will allow the service to be scaled up?

### Study procedures

#### Impact evaluation phase (randomised controlled trial)

Once enrolled, parents and carers will be randomised to the intervention or control group using the WMG-E enabled computerised randomisation. Study information for participants will only discuss general groupings to give parents and carers sufficient information to consent to the trial. Parents and carers in both groups will use the WMG-E platform to complete the demographic, primary and secondary outcome measures. Where needed, researchers will issue reminders to participants who have not completed all the baseline assessments and will assist those with issues of digital literacy to guide them through completion.

#### Intervention group

##### Main trial

After completing the baseline questionnaires, a digital service navigator at each site will direct families to the appropriate care pathways depending on their presentation for the different tiers (figure 3). The digital navigators will also provide ongoing support to families throughout their participation and monitor emerging or changing needs to ensure timely adjustment of referrals and care pathways where appropriate. Further, children identified as being developmentally vulnerable or families with complex mental health and social care needs will be highlighted for follow-up by an in-person service navigator (digitally in WA) who will link them with the most appropriate healthcare professional, such as a child and family health nurse or paediatrician. The in-person service navigator will provide a warm handover to the healthcare professionals, who will provide further assessments and appropriate support and referral pathways for children identified as having developmental concerns, as per usual care. Families identified as having financial difficulties or unmet psychosocial needs will be referred to support services (eg, financial counsellor). Families that screen positive for mental health symptoms will be linked with a psychologist for further assessment and mental health first aid, along with brief interventions and referrals. Tier allocation might be revised at the discretion of the service navigator or following multidisciplinary team case review, based on emerging needs or updated information.

**Figure 3:**
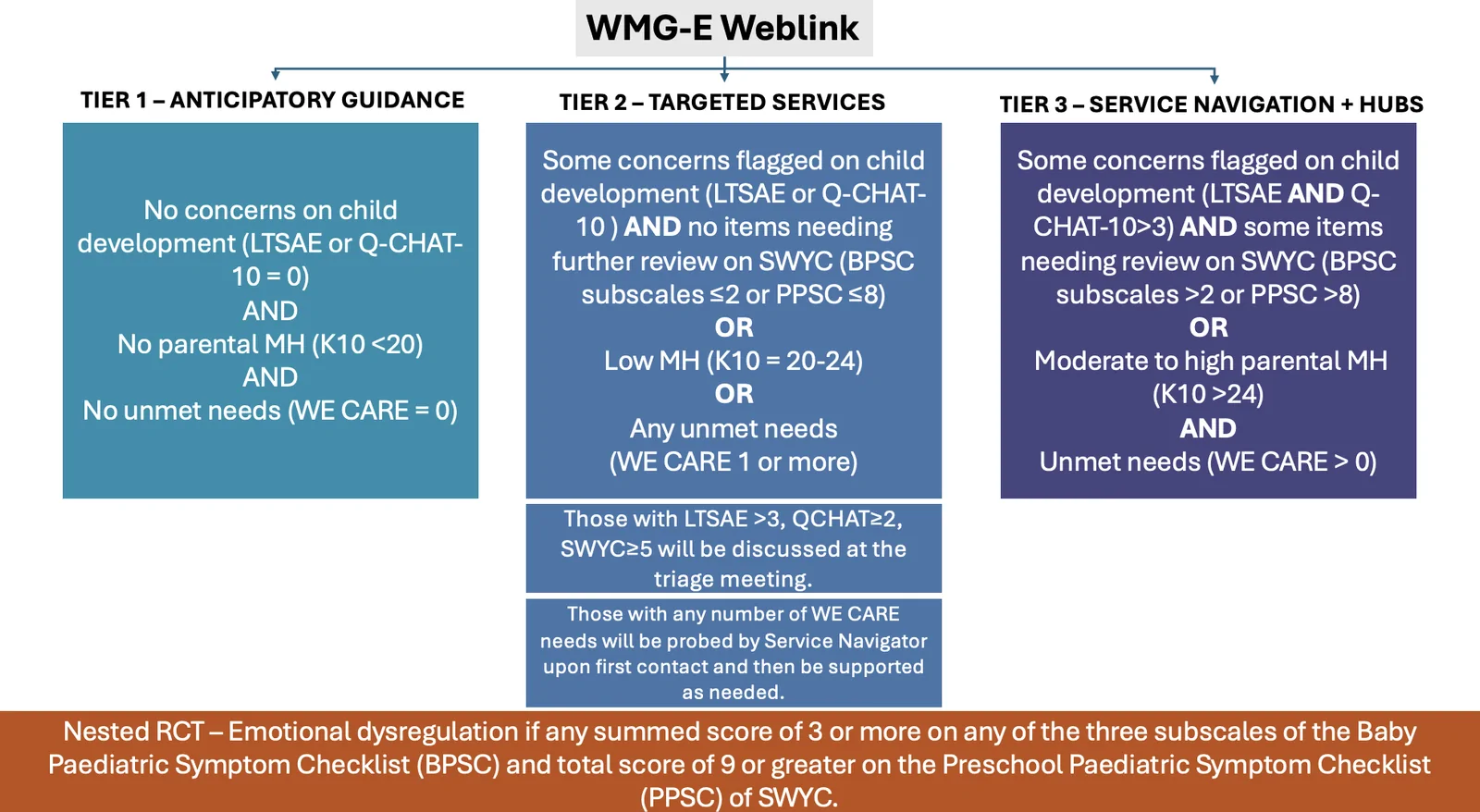
Care pathway based on the outcome measures. K10, Kessler Psychological Distress Scale–10; LTSAE, Learn the Signs. Act Early.; MH, mental health; Q-CHAT, Quantitative Checklist for Autism in Toddlers; Q-CHAT-10, Quantitative Checklist for Autism in Toddlers–10; RCT, randomised controlled trial; SWYC, Survey of Well-being of Young Children; WE CARE, Well-Being and Caregiver Assessment and Research Evaluation; WMG-E, Well-Being and Mental Health Evaluation.

##### Nested trial

The researchers will invite parents and carers who meet the eligibility criteria for the nested trial’s MTAM-EVP intervention to participate in the video programme. These parents and carers will receive a separate PISCF with details about the video programme intervention, and consenting participants will be enrolled into the nested trial.

Overall, the researchers will receive results from the screening measures. Participants allocated to the control group who are at risk of homelessness or acute crisis based on their questionnaire responses will be immediately linked with clinical support services readily available to all families via routine care. These participants will remain in the trial and be analysed according to their randomised allocation (intention to treat), with referral recorded as a protocol deviation.

### All participants

All participants will be issued with follow-up emails at 6, 12, 18 and 24 months asking them to complete the same questionnaires via the weblink again as part of their assessments. Parents and carers will receive a AUD 20 gift voucher for their time when they complete the questionnaires that are automatically emailed to consented parents at relevant timepoints.

### Implementation evaluation phase

If parents and carers consent to an interview, a researcher will contact them to arrange a time to conduct the interview via telephone or online platform (Zoom or Microsoft Teams). Interviewed parents and carers will receive a AUD 50 gift voucher for their time at 12 and 24 months in line with the remuneration and reimbursement guidelines for consumer involvement in health and medical research.

### Analysis plan

#### Impact evaluation phase

To compare outcomes between the intervention and control groups, an intention-to-treat analysis will be used with Fisher’s test for binary outcomes, χ2 method for categorical outcomes, non-parametric method (eg, Wilcoxon rank-sum test) and parametric methods (eg, t-test) for continuous variables and ordinal variables. As the outcomes will be measured repeatedly, linear mixed models will be used to model the outcomes over all time points, and the impact of the tiered care model on these outcomes will be examined at the 12-month and 24-month timepoints, controlling for the plausible confounders (eg, sociodemographic characteristics). R Studio software (version 2026.04.0+526) will be used to conduct the statistical analyses.

#### Implementation evaluation phase

Interviews will be recorded digitally, and verbatim transcripts will be analysed via an iterative thematic analysis framework using NVivo Pro V.12. Braun and Clarke’s six-phase method of thematic analysis will be used to ensure analytical rigour.^57^ The first phase involves familiarisation with the interview data, which requires immersion in the dataset through repeated listening to audio recordings alongside concurrent reading and re-reading of the transcripts. The second and third phases consist of deduction coding to the CFIR constructs and inductive coding for residual themes. Repeated patterns and meanings will be identified and organised into initial codes, and then broad themes and sub-themes will be generated. The fourth and fifth phases will involve within-case summaries and across-case comparisons. The fifth phase involves further development and refinement of the themes and sub-themes. In the sixth phase, final themes will integrate relevant extracts from participants’ transcripts with the guiding narrative to authentically convey their experiences.

#### Economic analysis and dynamic simulation modelling phase

The economic analysis will be conducted in three stages. First, implementation costs of the STARS for Kids model will be assessed, including the resources required to accurately identify and support needs, as well as the expected increase in parental engagement and uptake of services attributable to the intervention. Second, the economic value of the intervention will be evaluated from two perspectives. From a health sector perspective, the analysis will incorporate child developmental outcomes, service delivery costs, health utilities (EQ5D5L) and changes in acute service use (eg, emergency department presentations). From a societal perspective, the analysis will be broadened to capture longer-term benefits, including changes in productivity and the avoidance of other acute service use (eg, homelessness). This component will draw on secondary literature to model established relationships between study outcomes, for example, links between K10 categories and economic participation. A return-on-investment analysis will be undertaken to estimate the overall net benefit of the intervention, inclusive of cost savings. Third, longer-term costs and benefits will be modelled using a dynamic simulation model. This will create a computational representation of STARS for Kids, wider service system structures and outcomes of interest to project life course impacts. To inform model parameters (trajectories, outcomes and costs), evidence will be synthesised from several sources, including the trial results, other longitudinal datasets relating to child and adolescent development and estimates from the literature. The model will enable ‘what-if’ policy analyses to identify opportunities to further improve outcomes by, for example, investing in scaling-up STARS for Kids, synergistic interventions (eg, greater availability of referral services) and wider structural drivers of needs (eg, opportunities for employment) to ensure the needs of children and guardian needs are more fully met.

### Patient and public involvement

Parents/carers, service providers and community leaders were actively involved in the design and protocol developed for the conduct of this study through an 18-month codesign process across the three sites, with specific efforts to engage priority populations, including those from CALD, Aboriginal and Torres Strait Islander background, rural/regional and socio-economically disadvantaged communities. Codesign activities comprised semi-structured interviews, workshops, focus groups and culturally appropriate methods such as yarning. These informed the development and iterative refinement of the STARS for Kids model using a structured design thinking approach.^45^ Consumer and community representatives are embedded in the study governance, contributing to decision-making, implementation and knowledge translation.

At the Taree site, the codesign of the project and ongoing development of the trial are grounded in the NSW Aboriginal Health Ethics Guidelines,^58^ which provide a framework for conducting research that is culturally safe, respectful and community-centred. These guidelines emphasise five core principles - net benefit for Aboriginal people and communities; Aboriginal community control of research; cultural sensitivity; reimbursement of costs; and enhancing Aboriginal skills and knowledge - which collectively ensure that research is not only ethically sound but also meaningful and beneficial to Aboriginal communities. By embedding these principles into the research process, the project prioritises Aboriginal ways of knowing, being and doing, recognising the importance of cultural knowledge systems and community leadership in shaping both the design and implementation of the study.

Central to this methodology is the active involvement of local Aboriginal participants and community representatives at every stage of the research. An Aboriginal Advisory group, Ngarra Wakulda, meets monthly to inform decision-making and data management, ensuring that the research reflects local priorities, builds on existing strengths and responds to community-identified needs. This approach fosters trust, supports self-determination and ensures that outcomes are relevant and accessible. Additionally, the guidelines promote accountability and reciprocity, meaning that the research must deliver tangible benefits back to the community, whether through capacity building, knowledge sharing or improved health outcomes. Through this collaborative and culturally grounded approach, the project aligns ethical practice with genuine partnership and long-term community impact.

### Governance

The governance structure comprises an executive committee, providing overall strategic direction and oversight, supported by the investigators meeting for scientific leadership and the research and operations group for fortnightly coordination of day-to-day activities. A comprehensive community engagement strategy operates in parallel through consumer advisory groups and an Aboriginal Advisory group, providing lived experience and cultural guidance to ensure relevance and inclusivity. Knowledge translation committees convene quarterly at each site to promote research uptake and local implementation. The operational and technical work is advanced by monthly dynamic modelling and health economics working groups, which inform system design and evaluation, alongside site committees that manage local implementation and stakeholder coordination (figure 4).

**Figure 4:**
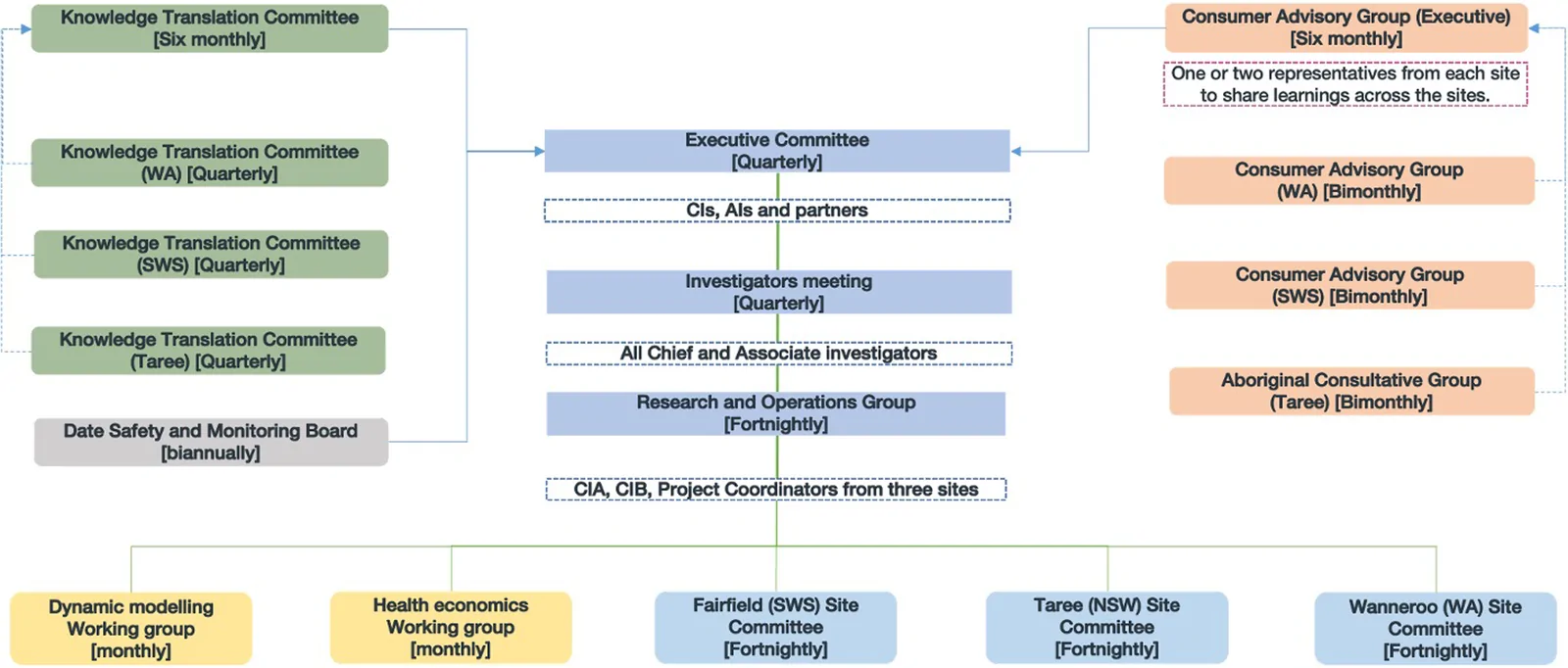
Governance structure. AI, Associated Investigators; CI, Chief Investigators; CIA, caregiver indirect assistance; CIB, caregiver indirect burden; NSW, New South Wales; SWS, South Western Sydney; WA, Western Australia.

### Ethics approval

The South Western Sydney Local Health District Human Research Ethics Committee approved this study for the SWS and Taree sites at NSW (2024/ETH01146), and Ramsay Ethics approved this study for the Wanneroo site at Western Australia (2025/ETH/0002). The trial has been registered in Australian New Zealand Clinical Trials Registry (ACTRN12624000806561). Site-specific research governance approvals were granted for all three sites. Given the substantial proportion of Aboriginal and Torres Strait Islander communities in Taree, an Aboriginal Health and Medical Research Council ethics application was approved specifically for the Taree site (2478/25). All aspects of the trial will adhere to the trial protocol (Version 9 or subsequent approved versions of the amendments) (online supplemental file 2) and Good Clinical Practice and Data Protection principles.

### Dissemination of findings

Data generated by the study will be disseminated through a range of channels, including written reports, peer-reviewed journal publications and presentations at relevant academic conferences and community forums. Access to study data by individuals other than the named researchers will only be permitted following consideration and agreement by the full research team and at the Taree site, the Ngarra Wakulda Aboriginal Advisory Group. Knowledge translation will be undertaken in close collaboration with study partners and advisors, who will support the sharing of findings with partner organisations and policymakers and inform decisions regarding potential scale-up and implementation of the programme. At least two peer-reviewed publications will be produced by the core researchers. Additionally, a lay summary of findings, accompanied by a graphical abstract and infographics, will be prepared and shared with participants who indicate an interest in receiving study results. Findings will also be disseminated to participants and community stakeholders through accessible formats, including plain-language summaries and community forums, to ensure broad and meaningful engagement with the research outcomes.

### Data management

All data will be deidentified for analysis and secured on password-protected computers on the University of New South Wales (UNSW) server. Only the deidentified data will be transferred from the sites to the UNSW researchers for data analysis. For each site, the dataset will exclude each participant identifying information and only include data alongside each participant's unique identifier. The data from each site to the UNSW team will be securely transferred through a NSW Health-approved e-health platform. Only the nominated researchers will have access to the data. If a participant withdraws from this study, they will be asked whether they would like their data removed from the dataset and deleted. All major issues arising from this study will be reported to the HREC with reasons for the issue and the associated implications.

Data from the RCT study will be retained for 25 years after the completion of this study in accordance with the South Western Sydney Local Health District (SWSLHD) Human Research Ethics Committee (HREC) requirements. Additionally, the interview recordings will be deleted as soon as they have been transcribed. The redacted interview transcripts and electronic data will be kept for 5 years and deleted at the end of this time. After that time, hardcopy materials will be destroyed by shredding, and secure electronic archives will be deleted. This is in accordance with the SWSLHD data management guidelines. Records will not be destroyed without the written consent of the coordinating principal investigator, if applicable. The coordinating principal investigator is responsible for informing researchers when these documents no longer need to be retained.

A small, independent Data Monitoring Committee (DMC) will be established and will convene every 6 months to oversee the trial data quality and participants’ safety. The committee will include experts in clinical trial methodology, health services research, statistics, mental health and child development. The DMC will review blinded outcome data and safety indicators to identify any unexpected benefits or potential adverse events (AEs) and will provide recommendations to the research team regarding whether recruitment should continue or be temporarily paused. The committee will operate under a formal procedures manual outlining its roles, responsibilities, processes and requirements for confidentiality.

### Adverse event reporting

Given the low-risk, service-delivery nature of the intervention, AEs are expected to be minimal and primarily related to psychosocial distress identified through screening. All AEs will be recorded by the principal investigator in REDCap, including onset, severity, outcome and assessed relationship to the intervention. Events will be followed until resolution where feasible. Significant safety issues and urgent safety measures will be reported in accordance with regulatory requirements to the sponsor, HREC and relevant governance bodies. Any identified psychosocial risks (eg, domestic violence, homelessness and severe mental health concerns) will be managed through existing clinical care pathways by service providers and the research team.

## Discussion

The STARS for Kids tiered care model aims to bridge the current fragmented and siloed service delivery by integrating the different health service elements with social and legal care at the consumer, service provider and health system level. By leveraging opportunistic service touchpoints, such as immunisation visits, playgroups, early childhood education and social service contacts, we will systematically reach children and families where they are in the community and facilitate access to wraparound health and social care tailored to their needs, recognising that barriers to engagement often arise from service accessibility and navigation challenges rather than family-level factors.^59^ Through targeted and integrated supports commensurate with needs, we will also overcome the current resource limitations, inflexible eligibility criteria, poor linkages and transitions. We will generate new evidence on a hybrid (in-person and digital) service delivery model for the early and accurate identification of children with developmental needs and will coordinate, re-purpose and systematise the existing service infrastructure to effectively intervene, including wraparound health and social care. By providing integrated supports using a proportionate universalism framework (universal services plus targeted supports commensurate with needs), we will overcome the current inequity in access to developmental services for children, particularly children from disadvantaged backgrounds.

## Supplementary material

10.1136/bmjopen-2026-122902 online supplemental file 1

10.1136/bmjopen-2026-122902 online supplemental file 2
