# Supplementary material for: Effect of strengths-based, tiered, accessible resources and supports (STARS) for Kids project, a codesigned hybrid service delivery framework to identify and support child development, parent mental health and psychosocial needs among parents and carers of children aged 6 months to 3 years: a randomised controlled trial protocol
Source: BMJ Open. 2026 Sep 29;16(9):e122902. doi: 10.1136/bmjopen-2026-122902 (PMC13630006; doi:10.1136/bmjopen-2026-122902)
Supplement: Supplementary file 1 [file bmjopen-16-9-s002.pdf]

Strengths-based, tiered, accessible, resources and supports (STARS for Kids): Effect of a co-designed blended service delivery framework to identify and support child development, parental mental health, and psychosocial needs among parents/carers of children aged 6 months to 3 years

Version Number	Date
Version 1	20/06/2024
Version 2	22/07/2024
Version 3	29/07/2024
Version 4	02/12/2024
Version 5	11/02/2025
Version 6	06/03/2025
Version 7	22/04/2025
Version 8	24/07/2025
Version 9	12/12/2025

Sponsor

This study is sponsored by 2021 Medical Research Future Fund Chronic Neurological Conditions (APP 2017709).

TABLE OF CONTENTS

1. PROJECT TEAM **3**

2. SUMMARY **10**

3. BACKGROUND AND RATIONALE **15**

4. STUDY AIMS/OBJECTIVES **17**

4.1 Overall Aim 17
4.2 Study Objectives 17
4.3 Study Hypotheses 17

5. METHODS **18**

5.1 STUDY DESIGN 18
5.2 PARTICIPATING SITES 19
5.3 Study participants 20
5.4 Recruitment, Consenting, and Randomisation 20
5.5 Intervention – STARS for Kids Tiered Care model 23
5.6 Data Collection 24
5.7 Study Procedures 28
5.8 Sample Size Calculation 30
5.9 Analysis Plan 30
5.10 Expected study duration 31

6. ETHICAL CONSIDERATIONS **32**

6.1 Study Procedure Benefits 32
6.2 Study risks and mitigation strategy 33
6.2.1 Participant distress 33
6.2.2 Participant safety 33
6.2.3 Staff safety 34
6.2.4 Adverse Event Reporting 34
6.2.5 Confidentiality and Privacy 35
6.2.6 Data Management, Storage, and Record Retention 36
6.2.7 Other ethical considerations 36
6.2.8 Governance and management 37
6.2.9 Data Safety And Monitoring Board 37
6.2.9 Conflict of interest 37

7. FUNDING **38**

8. DISSEMINATION OF FINDINGS **38**

9. REFERENCES **39**

10. APPENDIX **42**

1. PROJECT TEAM

OVERALL RESEARCH LEADS

Name: Valsamma Eapen
Position: Professor and Chair of Infant, Child, and Adolescent Psychiatry
Institution: University of New South Wales & Academic Unit of Child Psychiatry, SWSLHD
Role in the study: Coordinating and Site Principal Investigator (Fairfield, NSW site)
Email: v.eapen@unsw.edu.au

Name: Lauren Vaughan
Position: Manager, Children, Young People and Families
Institution: Hunter New England
Role in the study: Site Principal Investigator (HNELHD site)

Name: Aunty Kerrie Doyle
Position: Associate Dean
Institution: Western Sydney University
Role in the study: Principal Investigator (Aboriginal Lead for HNELHD site)
Email: AuntyKerrie.Doyle@westernsydney.edu.au

Name: Susan Woolfenden
Position: Professor of Community Paediatrics, Faculty of Medicine and Health and Director of Community Paediatrics at Sydney Local Health District (SLHD)
Institution: University of Sydney and SLHD
Role in the study: Investigator
Email: susan.woolfenden@health.nsw.gov.au

Name: Desiree Silva
Position: Professor of Paediatrics
Institution: University of Western Australia
Role in the study: Site Principal Investigator (Wannerroo, WA site)
Email: desiree.silva@telethonkids.org.au

Name: Amy Louise Finlay-Jones
Position: Head, Early Neurodevelopment and Mental Health team
Institution: Telethon Kids Institute
Role in the study: Investigator
Email: amy.finlay-jones@telethonkids.org.au

Name: Virginia Anne Schmied
Position: Professor of Midwifery
Institution: Western Sydney University
Role in the study: Investigator
Email: v.schmied@westernsydney.edu.au

Name: Ann Dadich

Position: Professor
Institution: Western Sydney University
Role in the study: Investigator
Email: A.Dadich@westernsydney.edu.au

Name: Raghu Lingam
Position: Professor, School of Population Child Health
Institution: University of New South Wales
Role in the study: Investigator
Email: r.lingam@unsw.edu.au

Name: Jane Rachael Kohlhoff
Position: Associate Professor
Institution: University of New South Wales
Role in the study: Investigator
Email: jane.kohlhoff@unsw.edu.au

Name: Daniel Lin
Position: Senior Lecturer, Discipline of Psychiatry and Mental Health
Institution: University of New South Wales
Role in the study: Investigator

Name: Ilan Barry Katz
Position: Professor in the Social Policy Research Centre
Institution: University of New South Wales
Role in the study: Investigator
Email: ilan.katz@unsw.edu.au

Name: Kenny Lawson
Position: Associate Professor
Institution: Western Sydney University
Role in the study: Investigator
Email: k.lawson@westernsydney.edu.au

Name: Andrew Page
Position: Professor
Institution: Western Sydney University
Role in the study: Investigator
Email: a.page@westernsydney.edu.au

Name: Jenny Anne Downs
Position: Associate Professor
Institution: Telethon Kids Institute
Role in the study: Investigator
Email: jenny.downs@telethonkids.org.au

Name: Shanti Raman
Position: Director, Department of Community Paediatrics
Institution: South Western Sydney Local Health District
Role in the study: Investigator
Email: shanti.raman@health.nsw.gov.au

Name: Adam Walker
Position: Senior Lecturer
Institution: University of New South Wales
Role in the study: Investigator
Email: a.walker@neura.edu.au

Name: Nicole Deen
Position: Lead, Capacity building
Institution: Australian Research Alliance for Children and Youth (ARACY)
Role in the study: Investigator

Name: Rebekah Grace
Position: Associate Professor
Institution: Western Sydney University
Role in the study: Investigator
Email: rebekah.grace@westernsydney.edu.au

Name: Susan Prescott
Position: Professor of Paediatrics
Institution: University of Western Australia
Role in the study: Investigator
Email: susan.prescott@uwa.edu.au

Name: James John
Position: Research Associate
Institution: University of New South Wales
Role in the study: Investigator
Email: james.john@unsw.edu.au

Name: Sara Cibralic
Position: Research Officer
Institution: University of New South Wales
Role in the study: Investigator

Name: Katarina Ostojic
Position: Research Fellow
Institution: University of Sydney
Role in the study: Investigator
Email: katarina.ostojic@sydney.edu.au

Name: Hayely Robinson
Position: Senior Project Officer
Institution: University of New South Wales
Role in the study: Investigator

Name: Maite Diez
Position: Research Assistant
Institution: University of Sydney
Role in the study: Investigator
Email: maite.diez@sydney.edu.au

Name: Sharon Goldfeld
Position: Professor
Institution: Murdoch Children's Research Institute
Role in the study: Investigator
Email: sharon.goldfeld@rch.org.au

Name: Karlen Barr
Position: Research Officer
Institution: SWSLHD
Role in the study: Investigator
Email: karlen.barr@health.nsw.gov.au

Name: Alison Barnes
Position: Research Officer
Institution: Western Sydney University
Role in the study: Investigator

Name: Michael Hodgins
Position: Senior Research Associate
Institution: University of New South Wales
Role in the study: Investigator
Email: michael.hodgins1@unsw.edu.au

Name: Paul Saunders
Position: Research Fellow
Institution: Western Sydney University
Role in the study: Investigator

Name: Rob Doyle
Position: Research Assistant
Institution: Western Sydney University
Role in the study: Investigator

Name: Katherine Boydell
Position: Professor
Institution: University of New South Wales
Role in the study: Investigator
Email: k.boydell@blackdog.org.au

South Western Sydney Team

Name: Grainne O'Loughlin
Position: Chief Executive Officer
Institution: Karitane
Role in the study: Investigator
Email: grainne.oloughlin@health.nsw.gov.au

Name: Sharlene Vlahos
Position: Director of Education and Business Development
Institution: Karitane
Role in the study: Investigator

Name: Wendy Geddes
Position: Director, Child, Youth & Family, Primary & Community Health, NSW Health
Institution: SWSLHD
Role in the study: Investigator

Name: Anna-Marie Kanaan
Position: Integrated Care Manager
Institution: Karitane
Role in the study: Investigator

Name: Erin Brandtman
Position: Program Lead System Navigator – Fairfield Integrated Child and Family Hub
Institution: Karitane
Role in the study: Investigator

Name: Lee Bratel
Position: NSW Community Programs Manager at Karitane
Institution: Karitane
Role in the study: Investigator
Email: leanne.bratel@health.nsw.gov.au

Name: Kate Short
Position: Senior Speech Pathologist
Institution: South Western Sydney Local Health District
Role in the study: Investigator

Name: Tom McClean
Position: Head of Research and Social Policy
Institution: Uniting
Role in the study: Investigator
Email: AuntyKerrie.Doyle@westernsydney.edu.au

Name: Kannan Kallapiran
Position: Child Psychiatrist
Institution: Queensland Health
Role in the study: Investigator

Taree (HNELHD) Team

Name: Bree Katsamangos
Position: Program Manager
Institution: Mission Australia
Role in the study: Investigator
Email: katsamangosb@missionaustralia.com.au

Name: Clare Brennan
Position: Director
Institution: First Steps Count Incorporated
Role in the study: Investigator
Email: clare.brennan@firststepscount.org

Name: Toni Carson
Position: Centre Leader
Institution: First Steps Count
Role in the study: Investigator
Email: toni.carson@firststepscount.org

Name: Luana Woodhouse
Position: Registered Nurse
Organisation: Biripi ACMC
Role in the study: Investigator

Name: Melissa Foster
Position: Connected Beginnings Case Worker

Organisation: Biripi ACMC
Role in the study: Investigator
Email: melissa.foster@biripi.au

Name: Sophie Nicholls
Position: Research Associate
Institution: University of Sydney
Role in the study: Investigator
Email: sophie.nicholls@unsw.edu.au

WESTERN AUSTRALIA TEAM

Name: Sini Borgen
Position: Research Officer
Institution: Telethon Kids Institute
Role in the study: Investigator

Name: Bec Young
Position: Research Officer
Institution: Telethon Kids Institute
Role in the study: Investigator

Name: Nina D'Vaz
Position: ORIGINS Project Biobank Manager
Institution: Telethon Kids Institute
Role in the study: Investigator

Name: Jackie Davis
Position: Co-Head, The ORIGINS Project
Institution: Telethon Kids Institute
Role in the study: Investigator

2. SUMMARY

**Study Title**	Strengths-based, tiered, accessible, resources and supports (STARS for Kids): Effect of a co-designed blended service delivery framework to identify and support child development, parental mental health, and psychosocial needs among parents/carers of children aged 6 months to 3 years
**Short Title**	Strengths-based, tiered, accessible, resources and supports (STARS) for Kids for parent/carers and their children.
**Aims/Objectives**	**Overall aim:** The aim of this study is to systematically improve screening and support for child developmental, parental mental health, and family psychosocial needs.<br><br>**Primary objective:** Ascertain the impact of the (STARS) for Kids tiered care model (versus control group) on developmental monitoring of children – i.e., parent-reported completion of child developmental checks (as outlined in the Personal Health Record Blue Book in New South Wales and Purple Book in Western Australia), and subsequent identification of, and support for childhood developmental vulnerability over the first five years of a child's life.<br><br>**Secondary objectives:**<br>1) Evaluate the impact of the tiered care model in supporting parental mental health and families' psychosocial needs.<br>2) Ascertain parents/carers and relevant stakeholders' (e.g., service providers, policymakers) experiences with the STARS for Kids program including perceived feasibility, satisfaction, and benefits as well as barriers including suggestions to improve the implementation of the program.<br>3) Investigate the economic value of the tiered care model, including dynamic simulation modelling (DSM) to project the long-term impact of the STARS for Kids program
**Study design**	Mixed methods Evaluation will be embedded within a randomised controlled trial (RCT).<br><br>For the **impact evaluation phase**, we will use a multisite randomised controlled trial (RCT) where participants will be randomised using the Watch Me Grow - Electronic (WMG-E) platform to either the STARS for Kids tiered care (intervention group) or enhanced usual care (control group). Further, through a **nested RCT**, the children in the intervention group with emotional dysregulation (i.e., poor ability to manage emotional response) as identified via WMG-E assessments and aged between 12-36 months via the Survey of Well-being of Young Children (SWYC) scores, will receive targeted parent intervention: My Toddler and Me Enhanced Video Program (MTAM- EVP) compared to the control group (Evaluation of the 'My Toddler and Me' video series, #ETH02734) compared to the control group.

	For the **implementation evaluation phase**, we will use qualitative interviews with a subset of parents/carers who participated in the study and relevant stakeholders such as service providers, policy makers to ascertain their experiences with the STARS for Kids program including perceived feasibility, satisfaction, and benefits as well as barriers including suggestions to improve the implementation of the program.<br><br>For the **economic analysis and dynamic simulation modelling phase,** we will use a quantitative approach using the primary and secondary outcome measures to model the impact of the STARS for Kids program for long-term outcomes.
**Study sites**	This study will be conducted at three sites: (i) Liverpool/Fairfield and Bankstown – culturally and linguistically diverse (CALD) communities in South Western Sydney (SWS); (ii) rural/regional and Aboriginal community in Taree (HNELHD) in NSW; and (iii) a low socioeconomic area of the Wanneroo catchment in WA.
**Eligibility criteria**	**Parents/carers**<br>- Participants will include parents/carers of children aged 6 months to 3 years at the three sites attending child and family health services (CFHS), refugee health services, supported playgroups, preschools and early childhood education centres, parenting groups, non-governmental organisations (NGOs), general practice (GP), special paediatric clinics, and other state and national child and family programs<br>- All participants will possess the ability to provide informed consent.<br><br>**Service Providers (For the implementation evaluation phase only)**<br>- Service providers providing care at relevant sites<br>- Ability to provide written informed consent.
**Intervention**	The STARS for Kids tiered care will be used as a *universal digital screening and ongoing monitoring tool* with pathways to universal and targeted tiered care services<br><br>**<u>Universal service:</u>** WMG-E platform will provide a digital front door to systematically identify the child's developmental, parental mental health, and family social care needs.<br><br>**<u>Tiered service approach:</u>** WMG-E platform will then provide pathways to services based on child/family complexity and needs.<br><br>***Additional resources (Tier 1):*** Parents/carers with mild concerns will be provided with anticipatory guidance, such as health and developmental literacy resources, tips, and awareness, etc.

	Targeted services (Tier 2): Parents/carers with child developmental concerns, or mild mental health issue will be connected via digital navigation to online/in-person services (e.g., Parent groups, Triple P online, etc).<br>***Service Navigation (Tier 3):*** Parents/carers with complex health and family psychosocial needs will be linked with an in-person Service Navigator who will connect them with relevant health (developmental assessments and interventions); and social care providers (e.g., place-based hubs, intensive parenting interventions (e.g., MTAM-EVP nested trial (#ETH02734)), programs to improve family and community connectedness) so that the families receive wraparound care with 'warm handover' to referred services and 'continuity of care' to ensure the referral needs are met.
**Control**	Parents/carers in the control group will receive enhanced usual care via WMG-E universal screening as well as additional resources (Tier 1).
**Data collection and follow-up periods**	<u>**Primary outcome measures - *Developmental surveillance***</u><br>Parent/carer-reported completion of child developmental checks (as outlined in the Personal Health Record Blue Book in NSW and Purple Book in Western Australia) at baseline and follow-up time points (6, 12, 18, and 24 months).<br><br><u>**Secondary outcomes**</u><br>***Developmental outcomes***<br>(i) Proportion of children identified as at developmental risk via Learn the Signs Act Early (LTSAE) (Appendix B) and (ii) the Quantitative Checklist for Autism in Toddlers (Q-CHAT-10) (Appendix C) will be completed to check for concerns about autism.<br><br>If developmental concerns are identified through the above questionnaires, the Paediatric Symptom Checklist (PSC) in the Survey of Well-being of Young Children (SWYC) (Appendix D) will be applied as a screening tool for overall emotional and behavioural concerns.<br><br>***Parental Mental health:*** Proportion of parents/carers identified as experiencing psychological distress and at risk of depression and anxiety symptoms, measured using the Kessler Psychological Distress Scale (K10) questionnaire (Appendix E).<br><br>***Unmet psychosocial needs:*** Proportion of parents/carers identified as having more than one unmet social need via the 'WE CARE' questionnaire (Appendix F). Questions regarding experiences of racism will be included (Appendix H), due to the relation between racism and health access barriers, inequities and unmet social needs.

	All the above measures will be self-reported at baseline, 6, 12, 18, and 24 monthsin the intervention group and at baseline, 12, and 24 months in the control group.<br><br>***Demographic information***<br>Demographic information will be collected through the WMG-E weblink. Data will be self-reported at baseline in both groups (Appendix A).<br><br>***Health care service use***<br>Services the family was linked with, and service satisfaction will be measured with the WMG-E service use questionnaire (Appendix G). Data will be self-reported at baseline, 6, 12, 18, and 24 months in the intervention group and at baseline, 12, and 24 months in the control group.<br><br>***Satisfaction***<br>A Patient Satisfaction Questionnaire (Appendix K) and NAVSAT questionnaire (Appendix L) will be used to determine satisfaction with the early identification of needs and the tiered care model to support the needs. Data will be self-reported at 12 and 24 months (intervention group only).<br><br>***Economic modelling:*** The EuroQol 5-Dimension Questionnaire 5-Levels (EQ-5D-5L) (Appendix I) and the Caregiver Indirect and Informal Care Cost Assessment Questionnaire (CIIQ) (Appendix J).<br><br>To minimise survey fatigue in the control group, we will administer a brief semi-structured questionnaire (Appendix M) at the 6- and 18-month midpoints. This will ask participants whether they have sought help for any needs identified at baseline and whether they encountered any barriers, rather than requesting them to complete the full set of questionnaires again.<br><br>***Nested trial measures:*** The nested trial measures (Appendix N-Q) and analysis plan can be found in the nested trial protocol (Evaluation of the 'My Toddler and Me' video series, #ETH02734).<br><br>***Qualitative interviews***<br>Semi-structured interviews will be conducted to gather the experiences of accessing the STARS for Kids program using the Consolidated Framework for Implementation Research (CFIR) five implementation domains evaluating the acceptability, adoption, appropriateness, fidelity (use), coverage and sustainability. Data will be collected at 12 and 24 months in both groups.

**Planned sample size**	**Impact evaluation phase:** Based on our pilot data, a sample size of 420 (140 per site) will give us 80% power to detect increase in developmental checks from 30% to 60% at a 5% significance level and will allow for 40% attrition.<br><br>**Implementation evaluation phase:** Approximately 30 families who participated in the study (15 from the intervention group and 15 from the control group) and 30 stakeholders (e.g., service providers, policy makers).
**Analysis considerations**	**Impact evaluation phase:** To compare outcomes between the intervention and control group, we will use an intention to treat analysis: the Fisher's test for binary outcomes; Chi-square method for categorical outcomes; non-parametric method (e.g., Wilcoxon rank-sum test); and parametric methods (e.g., t-tests) for continuous variables and ordinal variables. As the outcomes will be measured repeatedly, multilevel regression analysis will be undertaken to examine impact of the tiered care model on outcomes over time, controlling for the plausible confounders (e.g., sociodemographic characteristics). The generalised estimating equations method will be used in the regression analysis considering potential clustering effect by site. Additionally, descriptive analysis will be undertaken with frequencies and proportions, by the research team using R software.<br><br>**Implementation evaluation phase:** Interviews will be recorded digitally and following transcription will be analysed via an iterative thematic analysis framework using NVivo Pro 12 software.<br><br>**Economic analysis and Dynamic simulation modelling phase:** Economic analysis will evaluate the cost of implementation; accurate identification/addressing of needs and change in parental engagement/uptake of services. Both a health sector perspective (e.g. incremental cost-effectiveness ratios (ICERs)) and a societal perspective (e.g. cost-benefit) will be estimated. We will use the primary and secondary outcome measures to and also conduct dynamic simulation modelling project the cost and benefits of STARS for Kids program over time.
**Study duration**	39 months from study enrolment to study completion with data analysis and dissemination.
**Other key ethical considerations**	Following this 'general' ethics approval, Aboriginal Health Medical Research Council (AH&MRC) Ethics approval will be sought for the Taree (HNELHD) site, since in this site the experience of Aboriginal people is an explicit focus of all or part of the research; data collection is explicitly directed at Aboriginal peoples; the information has an impact on one or more Aboriginal communities; and Aboriginal people are known, or likely, to be significantly over-represented in the group being studied (compared to the 3.4% of total NSW population).

3. BACKGROUND AND RATIONALE

Context of the problem

Every nine minutes, an Australian child is born at risk of developmental challenges (e.g., speech delay, autism) and at the start of school, one in five Australian children do not have the developmental skills to thrive (1). Access to timely support is hindered by the complex, poorly co-ordinated, and inefficient nature of the Australian child and family service system, which is characterised by fragmentation, duplication, and gaps. Such inequity in access to early identification and support was exacerbated by the COVID-19 pandemic due to service disruption. Moreover, children from priority populations (low income, regional/rural residence, Aboriginal and Torres Strait Islander, culturally and linguistically diverse (CALD)) can face multiple barriers to early identification. They are often identified late, often at or after the start of school (2, 3), missing critical opportunities for early intervention. These children may also be at higher risk for poor self-regulation, an important determinant of future adverse outcomes such as substance use (4, 5), mental health concerns (6), and physical health problems (7). Aboriginal and Torres Strait Islander people (and other priority populations) have experienced inequitable access to mainstream child and family services, due to a legacy of policies and service delivery models that have systemically excluded them from participation in a culturally safe way. This has affected their physical, emotional, spiritual and social wellbeing (8, 9). Furthermore, Aboriginal people have had fewer opportunities to contribute to the design and development of research models that effectively utilize the strengths of Aboriginal communities and ensure cultural safety (8, 9).

The 2020 Productivity Commission Report (10) called for regular developmental checks in early childhood, and the recent National Children's Mental Health and Wellbeing Strategy (11) highlighted the need for service integration to ensure an optimal start to life. The NSW Health First 2000 Days Framework (12) and the National Action Plan for the Health of Children and Young People: 2020-2030 (13) further illustrated the critical need for integrated care and support for marginalised populations. Further, our work has shown inequity in service access for early identification (14-21) and in research participation, with families from priority populations less represented in health systems research; hence, service innovations are not designed for their needs (22). This results in late identification and late intervention when co-morbidities and complexities have set in; but this is more expensive and less effective. Recent Australian data estimate the cost of late intervention at $15.2 billion due to the need for crisis services and high-intensity complex care (23).

Current evidence and knowledge gap

This study is informed by previous research. For example, a rapid review of digital solutions to enhance early detection and early intervention found clear time and follow-up efficiencies in the use of web-based developmental surveillance programs (24) as used in this study. A systematic review on digital health interventions (DHI) suggested there is a paucity of high-quality evidence to improve parents' mental health literacy, help seeking, or service use, reiterating the need to evaluate DHIs by using rigorous study designs and consistent measures (25). Hence, we propose using the WMG-E platform and health literacy/resources for parents. Additionally, systematic reviews on integrated hub models of care for early detection and intervention of developmental delay in disadvantaged communities showed improvements in

specific outcomes like parenting, child behaviour, and potentially child development. However, there is insufficient evidence on integrated hub-based models of care. This emphasises the need for rigorous research of such models with clear implementation evaluation (26); a key focus of this study. Our systematic review on emotion regulation (ER) showed that children on the spectrum have a different repertoire of ER strategies and rely more on others to regulate their emotions. This highlighted the need for specialised parenting supports and in this regard, treatments incorporating both parents and children have been found to improve children's ER abilities (27). Hence, we will incorporate a novel, strengths-based online parenting program, developed by our team, the MTAM-EVP, to children identified to be at high risk of emotional dysregulation aged between 12-36 months. Thus, this study will gather the evidence required to develop integrated digital solutions and service models to improve access to early identification of and intervention for child developmental needs, and family psychosocial needs across the whole health, early childhood education and social service system pathway. This will address an important need, given that extant research largely focuses on fragments on this pathway.

Proposed solution

We will implement and evaluate a co-designed, technologically innovative, and translational program called the Strengths-based Tiered Access to Resources and Supports (STARS) for Kids to systematically improve the quality and consistency of universal screening methods using opportunistic contacts; and the engagement/empowerment of parents for its uptake and use across three sites: a CALD community in South Western Sydney (SWS) and a rural/regional community in Taree in NSW (Biripi Country) and a low socioeconomic area of the Wanneroo catchment in WA. These sites were chosen as areas of significant disadvantage, with three suburbs from SWS and Wanneroo being featured in a national survey of the top 10 communities impacted by COVID-19 for children experiencing family employment stress (28). We have co-designed the essential elements of the STARS for Kids program with parents/carers and service providers (2022/ETH01781). Based on the initial rounds of co-design, we will implement and test the program, which will continue to undergo changes as per our ongoing co-design and consultation work with consumers and service providers.

By providing a tiered transdisciplinary care system, we will address the current limitations in traditional services that are disorder/discipline specific, narrow, and restricted with siloed approaches. Through targeted and integrated supports commensurate with needs, we will also overcome the current resource limitations, inflexible eligibility criteria, poor linkages, and transitions. We will generate new evidence on a blended service delivery model for the early and accurate identification of children with developmental needs and will co-ordinate, re-purpose and systematise the existing service infrastructure to effectively intervene, including wraparound health and social care. Also, by providing integrated supports using a proportionate universalism framework (universal services plus targeted supports commensurate with needs), we will overcome the current inequity in access to developmental services for children, particularly children from disadvantaged backgrounds.

4. STUDY AIMS/OBJECTIVES

4.1 Overall Aim

The aim of this study is to systematically improve the screening and support for child developmental checks, parental mental health, and family psychosocial needs. This will be achieved by implementing and evaluating the impact and cost-effectiveness of a co-designed, technologically innovative, and translational program called Strengths-based Tiered Access to Resources and Supports (STARS) for Kids.

4.2 Study Objectives

Primary objective: Ascertain the impact of the (STARS) for Kids tiered care model (versus control group) on developmental monitoring of children – i.e., parent-reported completion of child developmental checks (as outlined in the Personal Health Record Blue Book in NSW and Purple Book in Western Australia), and subsequent identification of, and support for childhood developmental vulnerability over the first five years of a child's life.

Secondary objectives:

1) Evaluate the impact of the tiered care model in supporting parental mental health and families' psychosocial needs.
2) Ascertain parents/carers and relevant stakeholders' (e.g., service providers, policymakers) experiences with the STARS for Kids program including perceived feasibility, satisfaction, and benefits as well as barriers including suggestions to improve the implementation of the program.
3) Investigate the economic value of the tiered care model, including dynamic simulation modelling to project the long-term impact of the STARS for Kids program

The STARS for Kids program comprises:

1) **Impact evaluation phase** – To evaluate the impact of the STARS for Kids tiered care model in improving child developmental, parental mental health, and family psychosocial outcomes.
2) **Implementation evaluation phase** – To ascertain parents/carers' and relevant stakeholders' (e.g., service providers, policymakers) experiences with the STARS for Kids program including perceived feasibility, satisfaction, and benefits as well as barriers including suggestions to improve the implementation of the program.
3) **Economic analysis and Dynamic Simulation modelling phase –** To evaluate the cost-and benefits of the STARS for Kids program, including both 'within-trial' impacts and modelled for longer-term outcomes.

4.3 Study Hypotheses

Primary Objective

Children of families receiving service via the STARS for Kids tiered care model will have significantly greater completion of their developmental checks compared to those allocated to the control group. There will be a 30%-60% increase in completion, compared to those allocated to control group.

Secondary Objectives

Families in the intervention group receiving services via the STARS for Kids tiered care model will experience greater improvements in the child developmental, parental mental health, and unmet psychosocial needs being identified and supported; and service satisfaction, compared to those allocated to control group in both short (2-year follow-up) and long term (via the DSM).

5. METHODS

5.1 STUDY DESIGN

For the **impact evaluation phase**, we will use a multisite randomised controlled trial (RCT) where participants will be randomised to either the STARS for Kids tiered care (intervention group) or enhanced usual care (control group). Further, through a nested RCT, children aged between 12-36 months identified to have emotional dysregulation (i.e., poor ability to manage emotional response) via the Survey of Well-being of Young Children (SWYC) scores (Baby Paediatric Symptom checklist scores of 3 or more on any of the three subscales or Preschool Paediatric Symptom checklist scores of 9 or more on the WMG-E will receive targeted parent intervention: MTAM-EVP as part of Tier 3 and this will be compared to control group (**Figure 1**). For the **implementation evaluation phase**, we will use qualitative interviews with a subset of parents/carers who participated in the study and relevant stakeholders such as service providers and policy makers to ascertain their experiences with the STARS for Kids program including perceived feasibility, satisfaction, and benefits as well as barriers including suggestions to improve the implementation of the program.

For **economic analysis and dynamic simulation and phase,** we will use a co-design phase to define the scope and cost/benefits to include and a quantitative approach using the primary and secondary outcomes measures to model the impact of the STARS for Kids program across the life course for children and guardians.

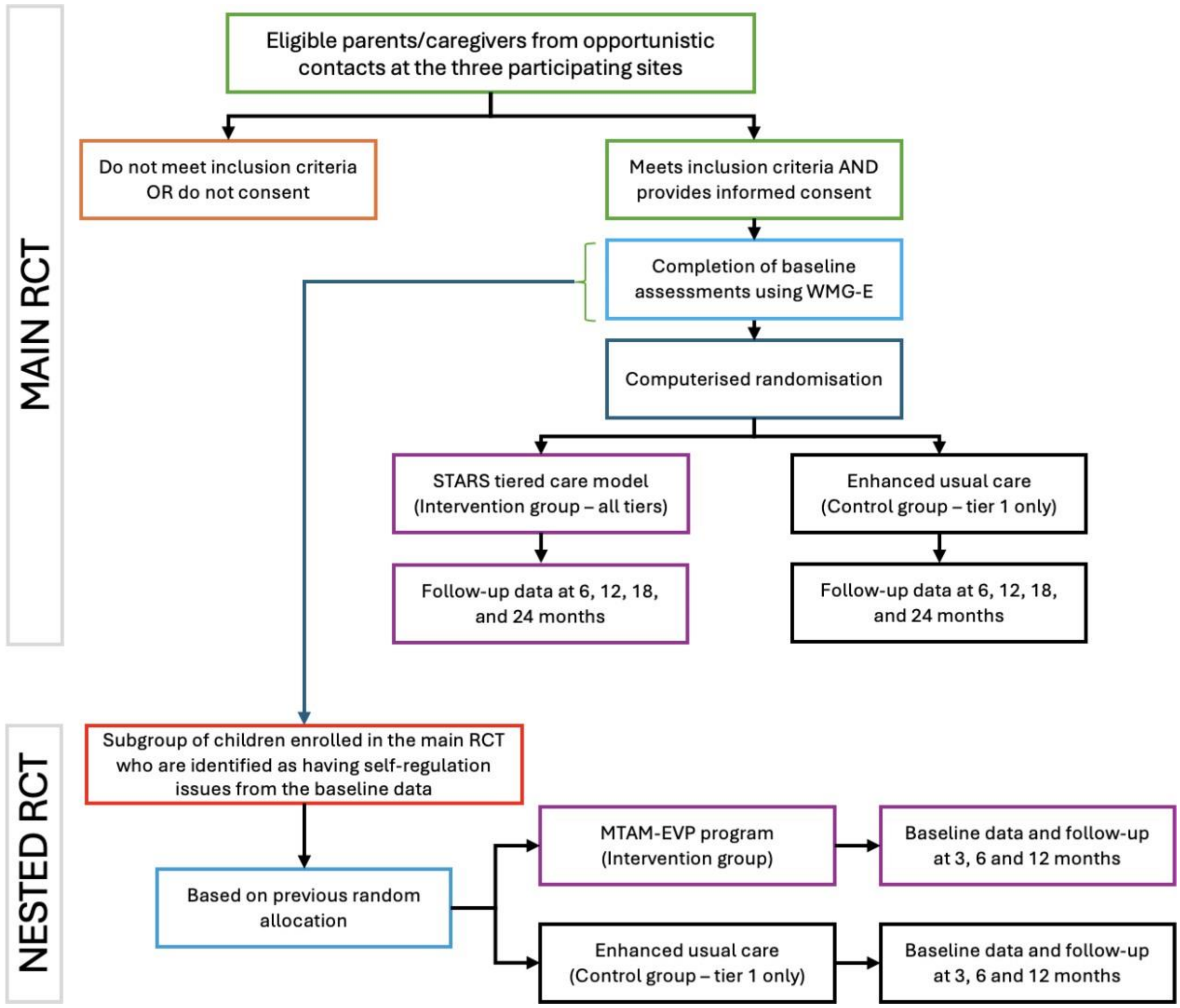

Figure 1. Study flowchart

5.2 PARTICIPATING SITES

The three sites include Liverpool/Fairfield and Bankstown in South Western Sydney (SWS); Taree (HNELHD) in NSW; and Wanneroo in WA. Within each site, we will use opportunistic contacts, child and family health services, refugee health services, supported playgroups, preschools and early childhood education centres, parenting groups, NGOs, GP practices, special paediatric clinics, and other state and national child and family programs for preschool children.

Site	Address	Site Principal Investigator	Phone	Email
Hunter New England LHD	4 Myall Avenue, Taree, NSW 2430	Lauren Vaughan	0477 395582	

Karitane Carramar Hub (SWS)	138-150 The Horsley Drive, Carramar, NSW 2163	Jane Kohlhoff	0297942300	jane.kohlhoff@unsw.edu.au
SWSLHD (Fairfield, Liverpool) (SWS)	Elizabeth Street, Liverpool, NSW 2170	Valsamma Eapen	0287383000	v.eapen@unsw.edu.au
Wanneroo Telethon Kids Institute (WA)	Northern Entrance, Perth Children's Hospital, 15 Hospital Ave, Nedlands WA 6009	Desiree Silva	0894009822	desiree.silva@telethonkids.org.au
**Chief Principal Investigator**<br>Professor Valsamma Eapen<br>Email: v.eapen@unsw.edu.au				
**Study Coordinators (study contact to be cc'd in all correspondence**)<br>Dr James John<br>Email: James.John@unsw.edu.au				

5.3 Study participants

Parents/carers

Inclusion Criteria – (i) Participants will include parents/carers of children aged 6 months to 3 years at the three sites attending child and family health services, refugee health services, supported playgroups, preschools and early childhood education centres, parenting groups, NGOs, GP practices, special paediatric clinics, and other state and national child and family programs (ii) Ability to provide informed consent.

Exclusion Criteria

- Parents/carers who do not have a child within the age range in the inclusion criteria
- Cannot be contacted via telephone or email
- Does not reside in geographical area of study.
- Less than 15 years of age at enrolment

Service Providers (For the implementation evaluation phase only)

- Service providers providing care at relevant sites
- Ability to provide written informed consent.

5.4 Recruitment, Consenting, and Randomisation

Impact evaluation phase (RCT)

Recruitment process

Parents/carers who meet the inclusion criteria at each site who attend child and family health services (CFHS), refugee health services, supported playgroups, preschools and early childhood education centres, parenting groups, non-governmental organisations (NGOs),

general practices (GP), special paediatric clinics, and other state and national child and family programs will be invited to participate via flyer/poster (physical paper or QR code) distributed by their trusted service providers on behalf of the researchers. The flyer/poster will contain the aim, objectives, anticipated outcomes, and researcher contact information. The flyers alongside other promotional material (e.g., short video) will also be advertised via a newsletters or email promotion to the appropriate health department staff directories, community centres, and as content for local Facebook posts and other appropriate social media platforms (e.g., Eventbrite). Given the substantial proportion of CALD communities in SWS site, flyers will be translated in Chinese, Arabic, Vietnamese. Given the possible high proportion of Aboriginal Torres Strait Islander participants in the Taree (HNELHD) site, the flyers will be co-designed with the Aboriginal Community Consultation Group at First Steps Count.

Parents/carers attending the above services at the study sites who are interested in the study can access the QR code on the flyer. Alternatively, staff at the above services can pass on contact details of parents/carers to the research team to follow up by email or telephone. Additionally, information sessions may also be held at relevant services (e.g., playgroups, community centre, etc) by the research team to go through details of the study to the participants and may clarify any queries arising about the study prior to consent. Participants who are interested will be initially assessed for eligibility by the research team. Following eligibility, the researchers will provide the participant information sheet and consent form (PISCF) in one of several ways depending on the family's preference and availability of time. The PISCF provides information about the aim and reason for research, its risks/benefits, and the voluntary nature of the study. Participants can elect to participate immediately or engage in a conversation with a nominated researcher (via telephone or email) and participate later. The PISCF states that if participants change their minds about joining the study, they are free to withdraw at any stage without consequence. Additionally, we will provide an alternative easy-read PIS which provides a simplified version of the aforementioned 'general' PIS as per the parents/carers' wish. Parents/carers can opt for either of the PISCFs and consent. If participants express that they are not interested in continuing participation, no further contact will be made in relation to the study. PISCF will be provided in several ways:

1) *Digital:* Parents/carers can access the QR code on the flyer which will prompt them to complete basic contact information and the eligibility criteria. Once they complete the form, they will be able to access the participant information sheet which will provide more details of the study. Concurrently, one of the research team will contact the parents/carers and re-assess eligibility and clarify any queries parents/carers might have before consenting.
2) *In-person:* Potential participants will be provided with a paper-based version of the PISCF/s during the face-to-face encounter or a digital version via QR code by the researchers at time of screening.
3) *Email*: If potential participants prefer to receive the PISCF via email or do not have time to be provided the paper-based PISCF, the researchers will send an email thanking them for their expression of interest and provide attachments, links via QR code (as applies) to the PISCF/s.

4) *Telephone:* If potential participants prefer to discuss the study and PISCF/s via telephone or do not have time to be provided the paper-based PISCF/s, the researchers will contact them by telephone. During the telephone call, the researchers will provide an explanation of the study, and if there is interest in participation, they will email the consent form or provide verbal consent via telephone.

We will provide either translated versions of PISCF or arrange interpretation services for participants who prefer to speak in a language other than English. Further, parents/carers with technological (e.g., internet access/issues) or literacy issues will be supported by the trusted service providers that the families are already engaged with and/or staff who are providing the flyer or introducing the parents to the study.

Consenting process

Participants can provide informed consent in several ways:

1) *In person:* Sign the hardcopy on paper or digital consent form via QR code on an electronic device at a face-to-face visit.

2) *Email contact followed by online consent:* The recruitment email will contain a full, plain language briefing of the study which can be accessed via the QR code of the flyers/posters. The email will explain that participation in the study is voluntary. The email will include a link directing potential participants to the online version of the PISCF via the Redcap® data weblink or by accessing the QR code. Potential participants will have to read the online PISCF and complete the digital consent form if they wish to participate. If the researchers do not hear back from the parent/carer within one week, a researcher will follow up twice to see if they have received the information and whether they have any questions. If the participant does not wish to participate, they will not be contacted again.

3) *Telephone contact followed by online consent:* When the researchers contact potential participants by telephone, an explanation of the study will be provided. If a potential participant is interested in participation and prefer to provide consent via online PISCF, they will be sent the aforesaid recruitment email with the flyer and the weblink or QR code to complete online PISCF.

4) *Telephone contact with verbal consent provided:* When the researchers contact potential participants by telephone, an explanation of the study will be provided. If a potential participant is interested in participation and prefers to provide verbal consent via telephone, they can provide verbal consent via telephone. This verbal consent process was successfully used by the team members, Woolfenden and Ostojic, in a project supporting migrant and refugee women with their adverse SDH in South-Western Sydney Local Health District (LHD) and Northern Sydney LHD (2020/ETH03295).

Parents/carers who would like to be involved in the nested trial will also be given the opportunity to read the PIS about the My Toddler and Me Enhanced Video Program (MTAM- EVP) intervention before consenting using the above methods.

Implementation evaluation phase (Qualitative subproject)
A random subsample of parents/carers enrolled in the RCT study as well as service providers will be approached for the qualitative subproject on the implementation evaluation. They will be provided with a PISCF providing information about the aim and reason for qualitative research, its risks/benefits, and the voluntary nature of the study, and asked to indicate whether they consent to being contacted for a more detailed interview (30-60 minutes) around the 12-month follow-up timepoint. Those who consent to being contacted will be invited to participate in the qualitative interviews at the 12 and 24-months' timepoints. Those who are interested to participate will be provided a detailed information sheet and consent form to provide consent via the above ways.

For both the phases, once eligible participants agree to participate, they will be able to either consent immediately or can take up to 1-2 weeks to consider consenting to participate. Every effort will be made to ensure it is safe for potential participants to autonomously consent. There will be no pressure to participate, and it will be made clear that declining to participate will have no consequence with health care provision.

Randomisation
Upon completion of the consent forms, participants will be randomised to either the intervention or control group via the WMG-E platform. Computerised randomisation software will be used to create a randomisation table via REDCap (a secure web application for building and managing online surveys and databases). Participants will be assigned randomly to each arm ensuring a 50:50 ratio.

5.5 INTERVENTION – STARS FOR KIDS TIERED CARE MODEL

Intervention group: The intervention group will receive the co-designed STARS for Kids tiered care model. This comprises the Watch Me Grow – Electronic (WMG-E) as a *universal digital screening tool that has been found to be effective* (29) *and acceptable* (30) *to families* (including translations and supported by CALD, out-of-home care, refugee, or other support workers, as needed) for the early identification of child developmental, parental mental health, and family psychosocial needs. WMG-E contains in-built logic to identify children at developmental risk at the point of care to aid shared decision-making. Based on the WMG-E screening results, resources and supports are provided as per the level of risk (see **Figure 2**). Results are automatically transmitted to parent/carer and the service provider who completes further assessments as needed. Once engaged, the platform issues automated reminders to the family, prompting them to take the developmental checks again at the next recommended ages and stages. The STARS for Kids tiered care intervention comprises universal and the full tiered care services as detailed below while the control arm will receive only Tier one services as part of 'enhanced usual care'.

Universal service: WMG-E platform as a digital front door to systematically identify the child's developmental, parental mental health, and family social care needs. This will also include automated reminders at the next age when the recommended developmental checks are due.

Tiered service approach: Based on complexity and needs.

Additional resources (Tier 1): Parent/carers with mild concerns will be provided with anticipatory guidance, such as health and developmental literacy resources, tips, awareness, etc.

Targeted services (Tier 2): Parent/carers with child developmental concerns or mild mental health issue will be connected via digital navigation with online/in-person services (e.g., Parent groups, Triple P online, etc) available at each site.

Service Navigation (Tier 3): Parents/carers with complex health and family psychosocial needs will be linked with an In-person Service Navigator who will connect them with relevant health (e.g. developmental assessments and intervention) and social care providers (e.g., place-based hubs, intensive parenting interventions (the MTAM-EVP program), strengths based approaches to increase family and community connectedness etc.) so that the families receive wraparound care with 'warm handover' to referred services and 'continuity of care' to ensure the referral needs are met.

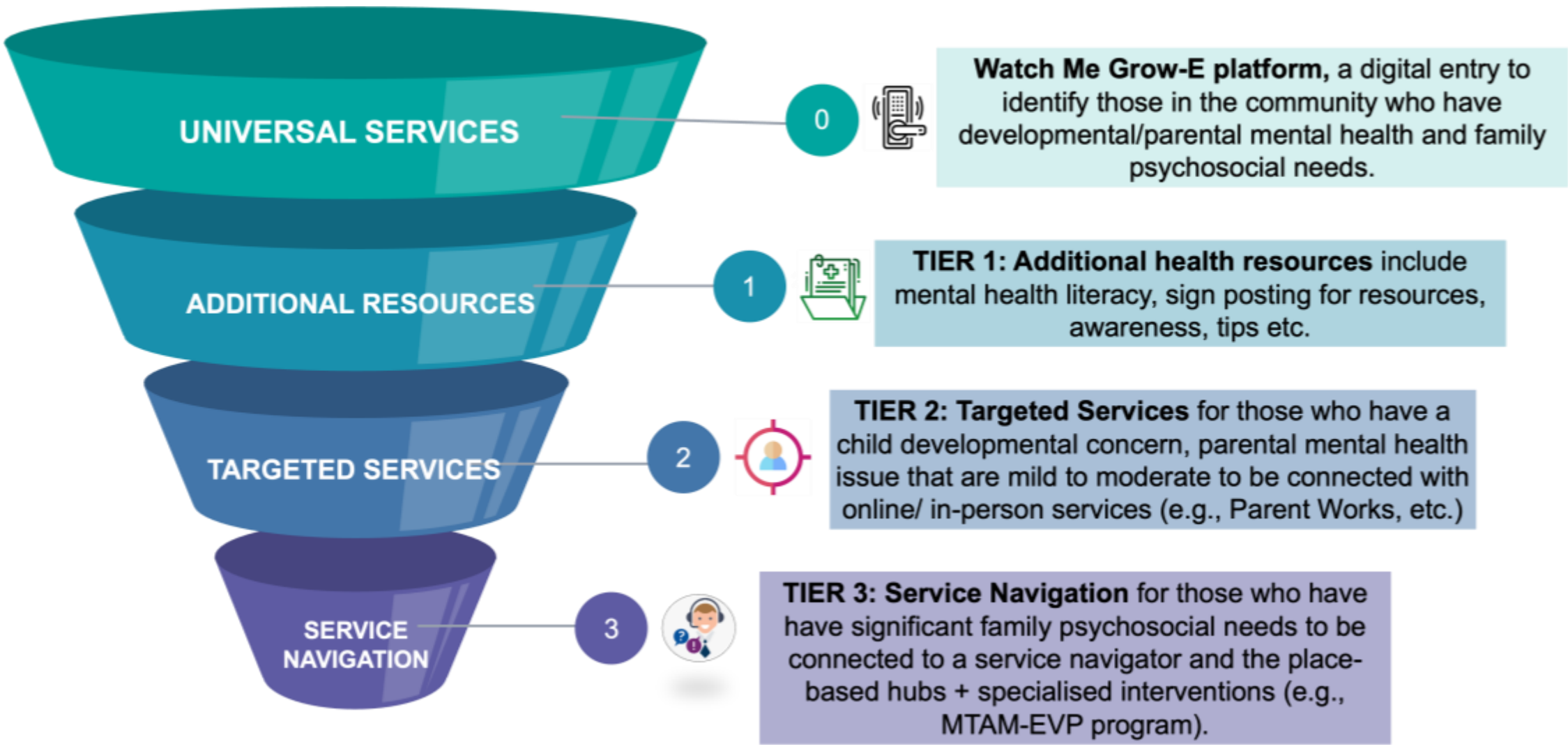

Figure 2. STARS for Kids Tiered Care Model

MTAM-EVP program: The MTAM-EVP program is aimed at improving self-regulation in toddlers (12-36 months) and promote parent/carer self-efficacy, mental health, and parenting skills.

Control group: Parents/carers in the control group will receive enhanced usual care via WMG-E universal screening as well as additional resources (Tier 1).

5.6 Data Collection

Demographic information: Child age and gender, as well as parent age, gender, ethnicity including if they identify as Aboriginal and/or Torres Strait Islander, country of birth, postcode, education, employment status, and family annual income. This will be self-reported only at baseline in both groups (**Appendix A**).

Measures

Primary outcome measures

Developmental checks: We will determine parents/carers-report of completion of developmental checks as outlined in the Personal Health Record Blue Book program in NSW or Purple Book in WA at baseline (i.e., completion of checks at ages before participation in the study). The Blue/Purple Book program recommends that health and developmental checks are completed at 6, 12, 18, 24, 36, 48, and 60 months. Parent/carer engagement with this recommendation at baseline will indicate alignment with Blue/Purple Book recommendations. At the follow-up timepoints (6, 12, 18, and 24 months), we will then track families to determine alignment with Blue/Purple Book recommendations. By comparing the intervention and control groups, we will determine whether the STARS for Kids program empowers parents to continue to engage in the completion of developmental checks at the follow-up time points as per the recommended ages compared to the control group. Our pilot data suggest the current engagement as 30%, which we expect to increase to 60% among families that access WMG-E.

Secondary outcome measures

Developmental milestones: We will use the Learn the Signs Act Early (LTSAE) (17) (**Appendix B**), which includes 'red flag' questions with 'yes', 'no', and 'not sure' response categories, for children aged 2 months to 5 years. LTSAE is used to monitor children's social**,** emotional, language**,** cognitive, and physical development. It can be completed in approximately 3 minutes. The WMG-E has a built-in algorithm that determines which LTSAE is done at what age based on the midpoint (i.e., if a child is under 9 months old, parents/carers will be requested to complete the 6-month LTSAE whereas if the child is more than 9 months old, a 12-month LTSAE will be completed). Secondly, we will also use the Quantitative Checklist for Autism in Toddlers (Q-CHAT-10) (31) (**Appendix C**) **-** a questionnaire to identify autism traits among children aged 16 to 35 months. The Q-CHAT-10 includes 10 questions and can be completed in approximately 3 to 5 minutes. Each question enquires about a specific behaviour, and parents/carers can answer according to the frequency of the behaviour the child displays.

If a concern is raised on the above screening questionnaires, a secondary screener, the Survey of Well-being of Young Children (SWYC) (32) will be used. SWYC is a comprehensive developmental and behavioural screening tool for children from birth to 5 years old. It assesses communication, gross motor skills, fine motor skills, problem-solving, personal-social skills, and emotional/behavioural health. Given several of the developmental milestones will be captured using LTSAE, we will only use SWYC's Baby and Preschool Paediatric Symptom Checklist (PSC) (**Appendix D**) to minimise research fatigue.

Parental mental health: Parental distress will be captured with the Kessler Psychological Distress Scale (K10) questionnaire (33) (**Appendix E**). The K10 assesses psychological distress over the past 4 weeks by querying how often the responder felt nervous or hopeless on a five-point scale (all the time, most of the time, some of the time, a little of the time, none of the time).

Unmet psychosocial needs: A modified 'WE CARE' questionnaire (34) (**Appendix F**), consists of six (yes/no) items querying: childcare, employment, homelessness, food security, education, and utilities. If parents/carers answer 'yes' to a question, a follow-up question appears asking whether they would like support in the nominated area (yes/no/maybe later).

Questions regarding experiences of racism will be included (**Appendix H**), due to the relation between racism and health access barriers, inequities, and unmet social needs.

All the above assessments will be collected at baseline, 6-, 12-, 18-, and 24-months follow-up timepoints via the WMG-E web-based platform in the intervention group and at baseline, 12, and 24 months in the control group.

Health care service use: Data on service use by the families will be collected in both groups (**Appendix G**) as self-report at baseline, 6-, 12-, 18-, and 24-months follow-up timepoints intervention group and at baseline, 12, and 24 months in the control group via the WMG-E web-based platform. Additionally, for both groups, service providers will be asked to complete a data log to record the number of children seen/discussed, the number of healthcare providers attending case discussions, the number of emails/telephone calls fielded, and the waiting time to access the services.

To minimise survey fatigue in the control group, we will administer a brief semi-structured questionnaire at the 6- and 18-month midpoints (**Appendix M**). This will ask participants whether they have sought help for any needs identified at baseline and whether they encountered any barriers, rather than requesting them to complete the full set of questionnaires again.

Satisfaction with the service: A patient satisfaction questionnaire (**Appendix K**) will be used to ascertain overall satisfaction whereas Navigation Satisfaction Tool (NAVSAT) (**Appendix L**). (35)will be used to specifically ascertain satisfaction with the service navigation in the intervention group. Data will be self-reported at 12- and 24-months follow-up timepoints via the WMG-E web-based platform.

Economic modelling: For economic modelling, The EuroQol 5-Dimension Questionnaire 5-Levels (EQ-5D-5L) (**Appendix I**) is a standardised instrument for measuring health-related quality of life. It consists of five dimensions: mobility, self-care, usual activities, pain/discomfort, and anxiety/depression, each rated on five levels of severity. It also includes a visual analogue scale (VAS) for overall health assessment. The EQ-5D-5L is widely used in clinical and economic evaluations to assess patient health outcomes and inform healthcare decision-making. Additionally, the Caregiver Indirect and Informal Care Cost Assessment Questionnaire (CIIQ) (**Appendix J**) is a tool designed to measure the indirect costs and informal care provided by caregivers. It evaluates factors such as time spent on caregiving, productivity loss, out-of-pocket expenses, and the impact on the caregiver's quality of life. This questionnaire helps quantify the economic and personal burden of caregiving for use in health economic studies and policy development.

Qualitative interviews

Semi-structured interviews (See Qualitative interview guide) will be conducted to gather parents/carers' and service providers' experiences of accessing the STARS for Kids program using the Consolidated Framework for Implementation Research (CFIR). The CFIR (36) will underpin the framing of the interviews and consider the barriers and facilitators for effective

implementation of the STARS for Kids tiered care model. Specifically, this will include feasibility, acceptability, appropriateness, adoption, fidelity, reach, and sustainability using Proctor et al implementation metrics (**Table 2**) (37). Data will be collected at 12 and 24 months in both groups.

Nested trial measures: Further details of the nested trial measures (**Appendix N-Q**) and analysis plan can be found in the nested trial protocol (Evaluation of the 'My Toddler and Me' video series, #ETH02734).

Table 2. Implementation evaluation metrics

Measure	Questions addressed by each implementation measure
Acceptability	Do consumers and service providers perceive the service as agreeable?
Adoption	To what extent do consumers and service providers perceive the service will be adopted in routine practice?
Appropriateness	Do stakeholders perceive the service as relevant and useful?
Fidelity	Is the service applied as intended?
Coverage	How many service users who were approached agreed to complete the service?
Cost	Are there any additional costs for services to implement the service?
Sustainability	What are the factors that will allow the service to be scaled-up?

The study schedule of data collection across timepoints is shown in **Table 3**.

Table 3. Study schedule of data collection

Measures	Baseline	End of RCT follow up (~3 months) Δ	6-month follow-up	12-month follow-up	18-month follow-up	24-month follow-up
Sociodemographic information	✓					
Parent-reported completion of child developmental checks	✓		✓	✓	✓	✓
Service use	✓		✓	✓	✓	✓
Developmental surveillance - LTSAE	✓		✓	✓	✓	✓
Autism screening - Q-CHAT-10	✓		✓	✓	✓	✓
SWYC - Baby and Preschool Paediatric Symptom Checklist	✓		✓	✓	✓	✓
Parental mental health (K10)	✓		✓	✓	✓	✓
Unmet psychosocial needs (WE CARE)	✓		✓	✓	✓	✓
Questions about racism	✓		✓	✓	✓	✓
EQ-5D-5L	✓		✓	✓	✓	✓
The Caregiver Indirect and Informal Care Cost Assessment Questionnaire (CIIQ)	✓		✓	✓	✓	✓

Participant satisfaction questionnaire*			✓	✓	✓	✓
Navigation Satisfaction Tool (NAVSAT)						✓
Qualitative interviews				✓		✓
**Nested trial**						
Patient-Reported Outcome Measurement Information System Early Childhood (PROMIS-EC) Wellbeing Measure ᐞ	✓	✓	✓	✓		
Multidimensional Assessment Profile of Disruptive Behavior (MAP-DB Infant-Toddler) – Temper Loss ᐞ	✓	✓	✓	✓		
Tool to Measure Parenting Self-Efficacy (TOPSE) – Three subscales only: 1) emotion & affection subscale; 2) empathy & understanding subscale; 3) play & enjoyment subscale ᐞ	✓	✓	✓	✓		
Coping with Toddlers Negative Emotions Scale (CTNES) ᐞ	✓	✓	✓	✓		

*Intervention group only
ᐞ Nested RCT participants only

5.7 Study Procedures

Impact evaluation phase (RCT)

Upon confirming eligibility and obtaining consent, parents/carers will be randomised using the WMG-E enabled computerised randomisation to the intervention or control group. However, study information for participants will only discuss general groupings to give parents/carers sufficient information to consent to the trial. Parents/carers in both groups will use the online WMG-E weblink to complete the demographic, primary, and secondary outcome measures at baseline. Where needed, a research staff will send reminders to those participants who have not completed all the baseline assessments and may help those with issues of digital literacy to guide them through completion. On completing the baseline questionnaire, a $20 Coles/Myer gift voucher will be given (either via postage or online) to parents/carers as a gift for their time spent in participation.

Intervention group - After completing the baseline questionnaires, a digital Service Navigator at each site will be allocated to direct families to different care pathways depending on their presentation for Tiers 1 and 2. A summary of the care pathway based on the outcome measures is shown in **Figure 3**. Further, children identified as being developmentally vulnerable or families with complex mental health and social care needs (refer to Figure 3 for thresholds) will be highlighted for follow-up by an in-person Service Navigator who will link them with the most appropriate healthcare professional, such as a child and family health nurse or paediatrician. The in-person Service Navigator will provide 'warm' handover to the healthcare professionals,

who will provide further assessments and appropriate support and referral pathways for children identified as having developmental concerns, as per usual care. Families identified as having financial difficulties or unmet psychosocial needs will be referred to support services (e.g., financial counsellor). Families that screen positive for mental health symptoms will be linked with a psychologist for further assessment and mental health first aid, along with brief interventions and referrals.

For those who meet the eligibility criteria for the My Toddler and Me Enhanced Video Program (MTAM- EVP) intervention via the SWYC cut-off scores on the WMG-E, the research team will get in touch with the parents to seek their interest in participating in the MTAM- EVP intervention via separate PISCFs which provide specific details about the MTAM- EVP intervention and only those who consent will be enrolled into the nested trial.

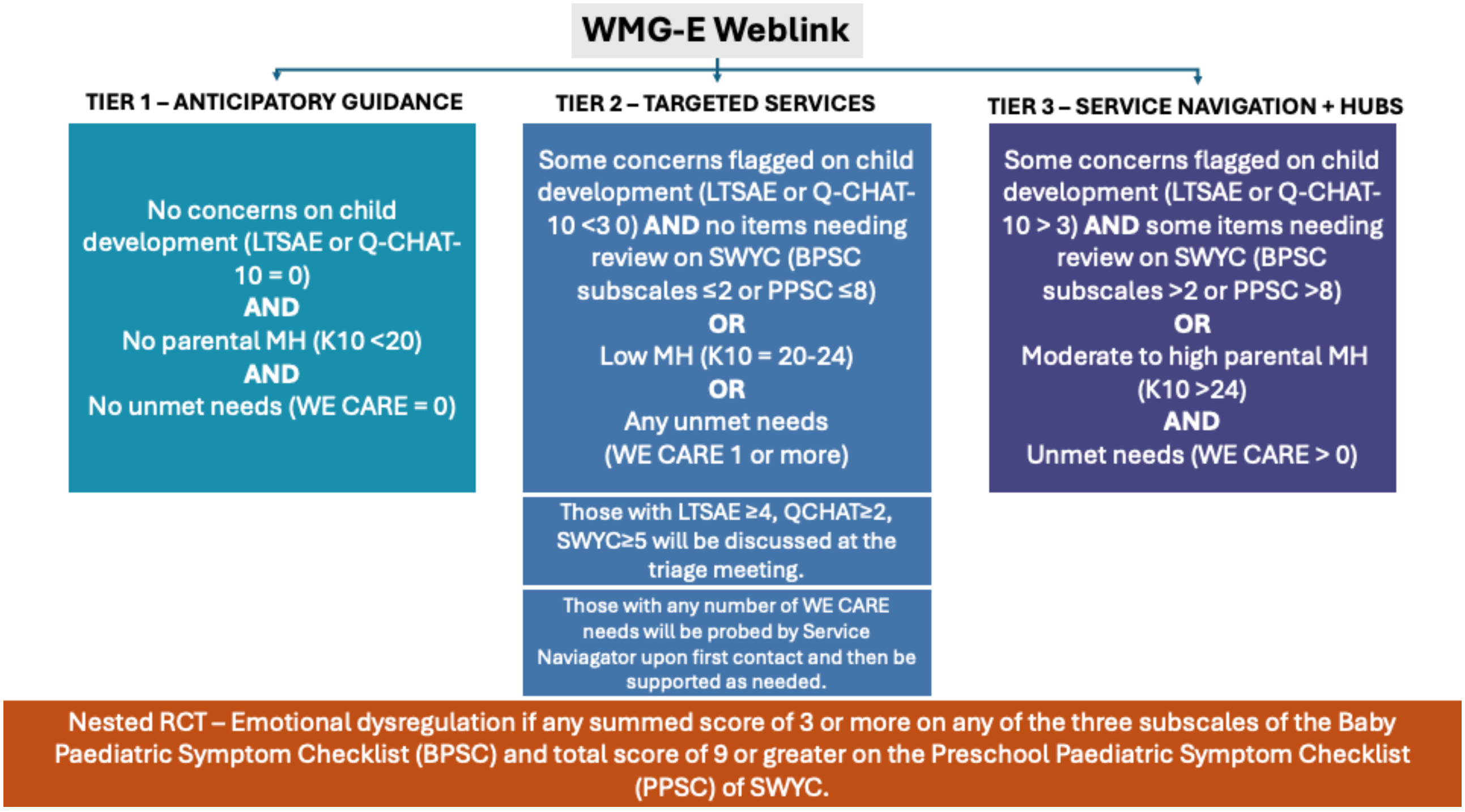

Figure 3. Care pathway based on the outcome measures

Tier allocation (Figure 3) may be revised at the discretion of the Service Navigator or following multidisciplinary team (MDT) case review, based on emerging needs or updated information.

Control group – After completing the baseline questionnaires, participants in the control group will receive 'enhanced usual care' via Tier 1 anticipatory guidance from the WMG-E weblink in addition to usual care from the organisations they already access.

The researchers will receive results from the screening measures. Participants allocated to the control group who are considered to be in 'acute crisis' based on their questionnaire responses will be immediately linked with clinical support services. The clinical support services are those readily available to all families via routine care.

All participants: All participants will be sent follow-up emails at 6, 12, 18, and 24 months asking them to complete the same set of questionnaires via the weblink again as part of their assessments. Families will receive a $20 Coles/Myer gift voucher for their time when they complete the questionnaires that are automatically emailed to consented parents at relevant timepoints.

Implementation evaluation phase (Qualitative subproject)
Once parents/carers consent to participate in the interview, a research officer will contact them to arrange a time to conduct the interview via telephone or online platform (Zoom or Microsoft Teams). Families will receive a $50 Coles/Myer gift voucher for their time in participating in the detailed interviews at both 12- and 24-month timepoints.

5.8 Sample Size Calculation

Impact evaluation phase: Based on our pilot data, and assuming a 30% increase in uptake of well-child checks in the intervention group (30% to 60%), 100 children (50 per group) per site (n=300) will provide 80% power to detect an increase with p value < 0.05 (two-tailed). Allowing for 40% attrition, 140 per group across 3 sites=420 is needed. For the nested RCT, a subgroup of children enrolled in the main trial who are identified as having self-regulation issues will form the study group; this is expected to be 20% (N~40 children in each site). Using generalized linear mixed models with two random effects (participants, site) and two fixed effects (treatment, time) approximately 90 participants are required to test the group x time interaction with a power of 0.80, assuming a small to moderate interaction effect (f = 0.15). Our feasible sample of 120 therefore allows for 25% loss to follow-up.

Implementation evaluation phase: Approximately 30 families who participated in the study (15 from the intervention group and 15 from the control group) and 30 stakeholders (e.g., service providers, policy makers). The sample size was established using the concept of 'information power' as articulated in Malterud et al's (38) guide to determining qualitative research sample size. Information power acknowledges that '…the more information the sample holds, relevant for the actual study, the lower' the number of participants required. Here, recruiting participants whose knowledge and lived-experience correlates specifically to the aims and focus of research, results in an information rich sample, as do other factors (38). In view of this, the recruitment of participants will result in high-level information power. Given our goal is to generate detailed, finely grained, qualitive data regarding the lives and experiences of these families and stakeholders, the sample size will be sufficient to meet the research aims.

5.9 Analysis Plan

Impact evaluation: To compare outcomes between the intervention and control group, we will use an intention to treat analysis: the Fisher's test for binary outcomes; Chi-square method for categorical outcomes; non-parametric method (e.g., Wilcoxon rank-sum test); and parametric methods (e.g., t-test) for continuous variables and ordinal variables. As the outcomes will be measured repeatedly, multilevel regression analysis will be undertaken to examine impact of the tiered care model on outcomes at the 12- and 24-month timepoints, controlling for the

plausible confounders (e.g., sociodemographic characteristics). The generalised estimating equations method will be used in the regression analysis considering potential clustering effect by site. Additionally, descriptive analysis will be undertaken with frequencies and proportions, by the research team using R software.

Implementation evaluation: Interviews will be recorded digitally and following transcription will be analysed via an iterative thematic analysis framework using NVivo Pro 12 software. Braun and Clarke's six-phase method of thematic analysis will be implemented to ensure rigour in the analysis process (39). The first phase identified by Braun and Clarke is familiarisation with the interview data. This involves immersion in the data by repeatedly listening to the digital recordings while re-reading and re-reading the interview transcripts. The second and third phases consist of identifying patterns and meanings, organising these into initial codes, and then generating broad themes and sub-themes. The fourth phase of analysis involves reviewing the data set to ensure themes are coherent and supported by the data and the fifth phase involves further development and refinement of the themes and sub-themes. In the sixth and final phase of analysis, final themes will integrate relevant extracts from participants' transcripts with the guiding narrative to authentically convey their experiences.

Economic analysis and dynamic simulation modelling phase: The economic analysis will be conducted in three stages. First, to assess the cost of implementing the STARS model to accurately identify and support needs, and the expected increase in parental engagement/uptake of services. Second, the assessment of economic value will be undertaken from both: (i) a health sector perspective and include child developmental outcomes, service costs, health utilities (using the K10) and changes in acute service use (e.g. ED presentations), (ii) a societal perspective will then widen the potential benefits to include, for example, productivity changes and avoidance of other acute services (e.g. homelessness) over time. This will rely on secondary literature regarding known relationships between study outcomes e.g. between K10 categories and economic participation. A return-on-investment analysis will evaluate the overall net benefit (inclusive of savings). Third, longer term costs and benefits will be modelled using a system dynamics model. This will create a digital representation of STARS, the wider service system and outcomes of interest to project life course impacts. To inform model parameters (trajectories, outcomes, and costs) evidence will be synthesised from several sources, including the trial results, other longitudinal datasets relating to the life-course and estimates from the literature. The model will enable 'what-if' policy analyses to identify opportunities to further improve outcomes by, for example, investing in scaling-up STARS, synergistic interventions (e.g. greater availability of referral services) and wider structural drivers of needs (e.g. opportunities for employment) to ensure the needs of children and guardian needs are more fully met.

5.10 Expected study duration

39 months from enrolment to completion of data analysis and dissemination (see **Figure 4**).

Task	2024		2025				2026				2027		
	Q3	Q4	Q1	Q2	Q3	Q4	Q1	Q2	Q3	Q4	Q1	Q2	Q3

Recruitment and consenting													
Data collection – Baseline													
6-month follow-up													
12-month follow-up													
18-month follow-up													
24-month follow-up													
Dynamic simulation modelling													
Data analysis													
Dissemination													

Figure 4. Gantt chart with timeline of activities

6. ETHICAL CONSIDERATIONS

6.1 Study Procedure Benefits

The STARS for Kids tiered care model will bridge the current fragmented and siloed service delivery by integrating the different health service elements with social care at the consumer, service provider, and health system level. We will promote ***service system innovation*** (e.g., coordination and integration) and ***redesign*** (technology navigation) of existing resources to support the developmental needs of the 'whole child' and their families. We will use the innovative Watch Me Grow Electronic (WMG-E) platform, a digital developmental screening and monitoring tool that our team has developed and piloted. Participants will be informed that, by participating in this study, they might be more aware of the signs and symptoms of developmental disorders and delay, regardless of whether their child is identified as being at risk of developmental disorders. They will also be informed that this information and knowledge might benefit continued monitoring of their child's development or in dealings with other children, friends, or family members.

This study harnesses opportunistic service attendance, such as immunisation visits, playgroups, early childhood education, and social service contacts to: systematically reach all (including hard to engage) children; and deploy wraparound health and social care (40). The effectiveness data from the three diverse (multicultural, regional/rural, and low income) sites will have broad applicability to other comparable sites across Australia to address longstanding health inequities. As the health system can be difficult to understand and navigate (40), the tiered care model will be a gamechanger. It will involve the use of opportunistic contacts to engage and empower parents/carers and equitably identify developmental delay for all children. This will be of national and international relevance as the first ever blended (digital/in-person) program for the early identification of developmental delays and a tiered care to support children including strengths-based approaches to improve family and community connectedness. This will reduce inequities in developmental service access - a MRFF priority area, '*healthy start to life'*, in the context of a post-Covid service delivery reset.

6.2 Study risks and mitigation strategy

6.2.1 Participant distress

We do not anticipate major risks to participants who agree to participate. Nevertheless, it is possible that the questions in the questionnaires/assessments might raise issues that are uncomfortable, upsetting, or frustrating for participants. To address the risk of potential distress to participants:

1. The researchers will provide extra time with the family to support them regarding their distress and concerns following the completion of questionnaires/assessments and direct them to a child and family health service if they request assistance.
2. Contact details for a range of mental health support services and local services to support child development are indicated in the Participant Information Statement.
3. The Participant Information Statement states that participants can cease participation at any time.
4. The researchers include clinician researchers, trained to support participants who experience distress through a risk management framework. For instance, Prof. Eapen and Prof. Woolfenden have extensive experience working with families in similarly intricate situations for years across clinical, pastoral, and longitudinal settings.

6.2.2 Participant safety

The Participant Information Sheet indicates that all data and information provided to the researchers are confidential and will not be made available to other organisations. Researchers have an additional ethical responsibility to seek support for a participant if there are concerns about their safety or that of their children. If issues are disclosed beyond the study parameters, mandatory NSW (and WA relevant) Health policy directives will apply (e.g., family and domestic violence or child protection matters) and these will be managed as per current policies and practices within the local health districts. The child protection and domestic violence counselling teams are within the child, youth, and family service and are readily accessible to provide advice and support if issues are identified. As the researchers are all mandatory reporters, they will inform participants that they are not able to maintain confidentiality when it relates to the safety of the participant, parent/carer, the child/ren, the family, and the wider community. In such situations, the researcher would discuss this with the participant in private. These obligations are detailed in the Participant Information Sheet.

Researchers have an additional ethical responsibility to seek support for a participant if there are concerns about their safety and these will be managed as per current policies and practices within LHDs. The Child Protection and Domestic Violence Counselling teams both sit within the Child, Youth & Family Service and so are readily accessible to provide advice and support if any issues are identified. As the research team are all mandatory reporters, they will inform participants that they are not able to maintain confidentiality when it relates to the safety of the participant, parent, the child/ren, the family, and the wider community. In such situations the project officer would discuss this with the participant in private. These obligations are detailed in the PISCF.

The researchers will adhere to the relevant NSW or WA Health documentation, including but not limited to:

- Mandatory Reporting Guide, Child Protection and Domestic Violence guidelines
- Complaint Management Policy
- Incident Management Policy
- Code of Conduct – NSW/WA Health
- Privacy Manual NSW/WA Health

6.2.3 Staff safety

Researchers will be trained to respond appropriately to participants and take measures to ensure participant confidentiality and safety. Training will include the skills required to put parents/carers at ease, ensure that participants are not pressured to answer questions they do not wish to, and that they do not feel judged or blamed for their choices or feelings about their care. Researchers will debrief as a team weekly, and with the Principal Investigators at the site, as required. The working group will guide and inform the evaluation strategy, including culturally safe recruitment and data collection procedures.

6.2.4 Adverse Event Reporting

The following definitions describe safety events that require expedited reporting to stakeholders including the sponsor, researchers, HREC, and the local governance office:

- Significant safety issue (SSI): a safety issue that could adversely affect the safety of participants or materially impact on the continued ethical acceptability or conduct of the trial. Comment: An SSI is a new safety issue or validated signal considered by the Sponsor in relation to the intervention that requires urgent attention of stakeholders. This might be because of the seriousness and potential impact on the benefit-risk balance of the intervention, which could prompt regulatory action and/or changes to the overall conduct of the clinical trial, including the monitoring of safety and/or the administration of the intervention.
- Urgent safety measure (USM): a measure required to be taken in order to eliminate an immediate hazard to a participant’s health or safety. Note: This is a type of SSI that can be instigated by either the investigator or sponsor and can be implemented before seeking approval from HRECs or institutions.
- Adverse events (AE) are considered any untoward medical occurrence in a patient or clinical trial participant administered the intervention, which does not necessarily have a causal relationship with this treatment.

For the purposes of this study the PI will be responsible for recording all adverse events (AE), regardless of their relationship to the intervention. The AE will be described in the source documents (e.g. visit notes) and captured via REDCap® and will include:

- A description of the AE.
- The onset date, duration, date of resolution.
- Severity (mild, moderate or severe – what is the impact on the participant’s daily life?).

- Serious Adverse Events (e.g. significant distress from completing the survey especially about the social care needs).
- Any action taken, (e.g. treatment, follow-up tests).
- The outcome (recovery, death, continuing, worsening).
- The likelihood of the relationship of the AE to the intervention (Unrelated, Possible, Probable, Definite).

Changes in the severity of an AE will be reported. AEs characterised as intermittent will be documented for each episode. All AEs will be followed to adequate resolution, where possible.

All AEs will have their relationship to the trial intervention assessed by the PI (with advice from the Research and Implementation oversight committee as needed) who will evaluate the AE based on temporal relationship and clinical judgment. The degree of certainty about causality will be graded using the categories below. The relationship of the event to the intervention will be assessed as follows:

- Unrelated: there is no association between the trial intervention and the reported event. AEs in this category do not have a reasonable temporal relationship to exposure to the intervention or can be explained by a commonly occurring alternative aetiology.
- Possible: the event could have cause or contributed to the AE. AEs in this category follow a reasonable temporal sequence from the time of exposure to the intervention and/or follow a known response pattern to the intervention but could also have been produced by other factors.
- Probable: the association of the event with the trial intervention seems likely. AEs in this category follow a reasonable temporal sequence from the time of exposure to the intervention and are consistent with the known action of the intervention, known or previously reported adverse events related the intervention, or judgement based on the investigator's clinical experience.
- Definite: the AE is a consequence of administration of the trial intervention. AEs in this category cannot be explained by progression of the condition, other concurrent illness or psychosocial problems, medication, or other factors. Such events may be widely documented as having an association with the intervention or that they occur after rechallenge.

If domestic violence, financial crises, homelessness, severe mental health, child protection concerns or other urgent needs are identified through the psychosocial screen, these will be managed through existing clinical pathways by the child and family health nurse and the research team.

6.2.5 Confidentiality and Privacy

The researchers are responsible for ensuring the accuracy, completeness, legibility, and timeliness of the data reported. All source documents will be completed in a neat, legible manner to ensure accurate interpretation of data (i.e., within an electronic and on-site physical study binder file). The screening data, and any information provided in the expression of interest will be kept confidential. All participants (eligible and in-eligible) will be allocated a unique identifier code (a Participant Identification Number, or ID) by the project coordinator, and used throughout the course of the study. Any identifying information will be anonymised and de-identified. The site research team and Service Navigator will have identifiable

information of the participants who they directly engage with during screening and intervention delivery.

6.2.6 Data Management, Storage, and Record Retention

All data will be de-identified for analysis and secured on password-protected computers on the UNSW server. Only the de-identified data will be transferred from the sites to the UNSW research team for data analysis. For each site, the dataset will exclude each participant identifying information and only include research data alongside with each participant unique project ID. The data from each site to the UNSW team will be securely transferred through a NSW Health-approved e-health platform. Only the nominated researchers will have access to the data. In the unlikely event that a participant wishes to withdraw from this project, they will be asked whether they would like their data removed from the dataset and deleted. All major issues arising from this study will be reported to the HREC with reasons for the issue and the associated implications.

Data from the RCT study will be retained for 25 years after the completion of this research in accordance with the SWSLHD HREC requirements. Additionally, the audio recordings of the in-depth interviews will be deleted as soon as they have been transcribed. The redacted in-depth interview transcripts and electronic data will be kept for 5 years and will be deleted at the end of this time. After that time, hardcopy materials will be destroyed by shredding, and any secure electronic archives will be permanently deleted. This is in accordance with the SWSLHD data management guidelines. Records will not be destroyed without the written consent of the Coordinating Principal Investigator, if applicable. It will be the responsibility of the Coordinating Principal Investigator to inform researchers when these documents no longer need to be retained.

Professor Valsamma Eapen at the Faculty of Medicine and Health of the University of New South Wales will be responsible for the deidentified data storage, security and retention of the data. Electronic data will be stored on a password-protected secure location on UNSW OneDrive. Any paper printouts from the study will be stored in a locked filing cabinet located at the Discipline of Psychiatry and Mental Health, Level 3, Australian Graduate School of Management (AGSM) Building, UNSW. Printouts of results will be shredded prior to disposal. The questionnaire data will be held securely in UNSW REDCap servers that are password protected. All operational trial data (e.g. hard copies of flyers, ethics documentation, etc) will be stored electronically on SWSLHD password-protected network drives and only accessible to a small number of Chief Investigators. No external personnel will have access to the data.

For data collected via the WMG web tool, REDCap will be used to collect data, and the built-in audit trail will allow administrators to be able to determine all the activity and all the data viewed or modified by any given user.

6.2.7 Other ethical considerations

An ethics application will be submitted to the Aboriginal Health & Medical Research Council of New South Wales (AH&MRC) following approval of this 'general' ethics application to specifically cover data collection at the Taree site given that the experience of Aboriginal

people is an explicit focus of research at the Taree site with data collection explicitly directed at Aboriginal peoples; the information has an impact on one or more Aboriginal communities; and Aboriginal people are known, or likely, to be significantly over-represented in the group being studied (compared to the 3.4% of total NSW population). There is already an Advisory Group formed at the Taree site under the guidance of CI Aunty Kerrie Doyle.

6.2.8 Governance and management

A steering committee consisting of the CI/AI team will meet quarterly along with project staff to oversee the operational aspects of the study. Further, a research operation group consisting of project coordinators at each site will meet on a fortnightly basis to update on the research activities. A management Committee with representatives from the partner organisations along with additional experts co-opted to the project and stakeholders including consumer representatives (e.g. parents) will meet quarterly to provide oversight and to consider all the available evidence in order to develop/refine study protocols and make recommendations.

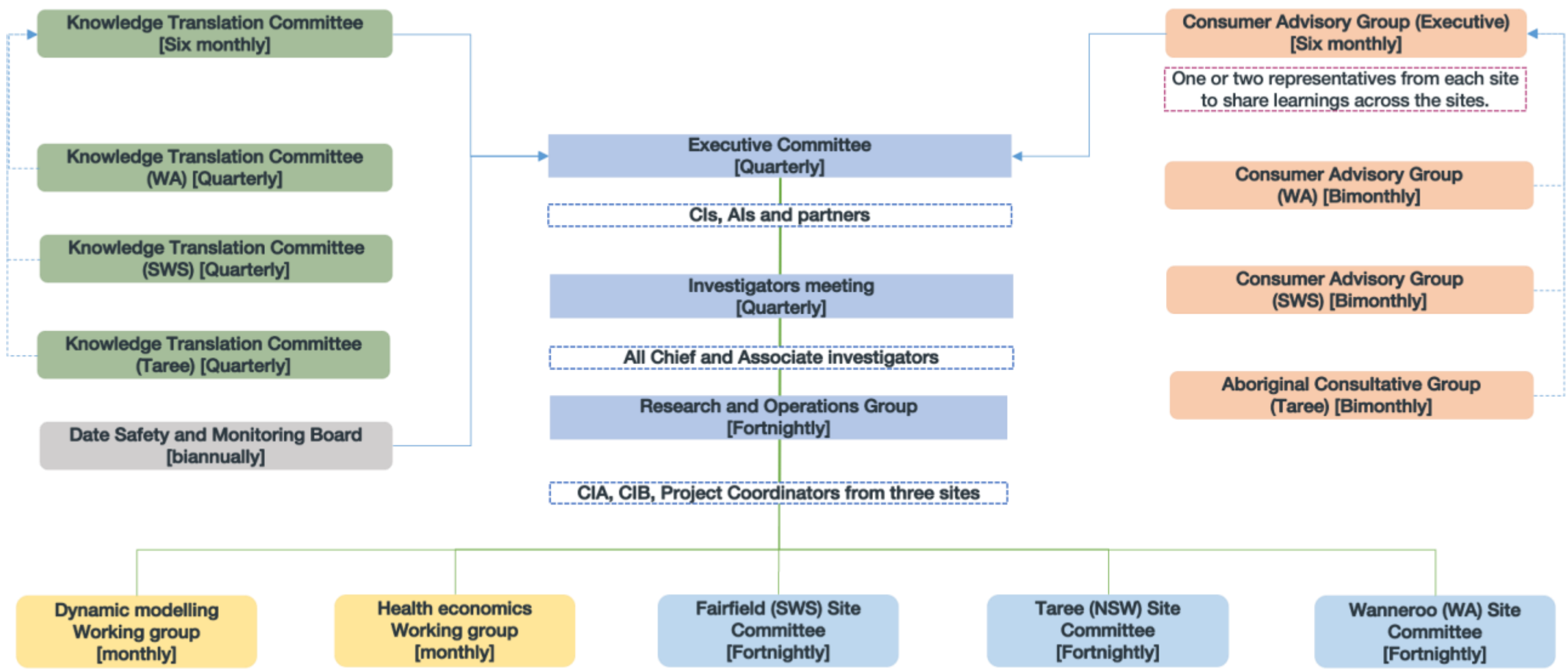

Figure 5. Governance structure

6.2.9 Data Safety And Monitoring Board

A small, independent Data Monitoring Committee (DMC) will be established and will meet once every six months to monitor the quality of the trial data and safety of research participants. DMC membership will consist of experts in clinical trial conduct in health services research, statistics, mental health, and child development. DMC will monitor blinded response variables and safety outcomes for any unexpected benefits or potential adverse events and provide reports to the researchers on recommendations to continue or temporarily halt recruitment to the study. The committee will be governed by an operating procedures manual that will outline their responsibilities, procedures, and confidentiality.

6.2.9 Conflict of interest

While it is acknowledged that both Principal Investigators hold public health roles, these positions are not directly linked to the services that will interface with this research. To mitigate any potential conflicts of interest in service providers also being researchers involved with

recruitment and data management, the research team will be employed by the UNSW at the Karitane Hub site and USYD at the Taree Hub site sites and the mixed methods researcher by USYD. Their role will be to work directly with local services to recruit participants, collect and analyse data. The in-person Service Navigator who are part of the intervention will be employed by the relevant sites.

Oversight of the research will be governed at three levels as outlined in the protocol (Project Governance). Members at each level of governance are from a range of backgrounds (i.e., frontline clinical and non-health staff, managers, researchers, and policy officers) and affiliated with other organisations outside of where the Principal Investigators hold positions (e.g. HNELHD, SWSLHD, Joondalup Health Campus, University of Sydney, Mission Australia, Western Sydney University, University of Western Australia, First Steps Count, Karitane).

7. FUNDING

This study is sponsored by 2021 Medical Research Future Fund Chronic Neurological Conditions (APP 2017709).

8. DISSEMINATION OF FINDINGS

Data obtained for the study will be published in reports and refereed journals, and presented at appropriate conferences and community fora. Access by individuals other than the named researchers will only be permitted after consideration and agreement by all the remaining researchers. Knowledge translation requires the study partners and advisors who will share findings with the partner organisations and policymakers to consider if and how to implement the program at scale. We intend to produce at least two papers (e.g., protocol, main findings) for peer-review publication, written by core research and implementation team. Further, a lay summary of the results with a graphical abstract and infographics will also be sent to those participants who are interested.

9. REFERENCES

1. Australian Early Development Census. Australian early development census national report 2015: A snapshot of early childhood development in Australia. Canberra, ACT: Department of Education and Training; 2015.
2. Sheldrick RC, Merchant S, Perrin EC. Identification of developmental-behavioral problems in primary care: a systematic review. Pediatrics. 2011;128(2):356.
3. Walkup JT, Stossel L, Rendleman R. Beyond rising rates: personalized medicine and public health approaches to the diagnosis and treatment of attention-deficit/hyperactivity disorder. Journal of the American Academy of Child and Adolescent Psychiatry. 2014;53(1):14-6.
4. Dishion TJ, Connell A. Adolescents' Resilience as a Self-Regulatory Process: Promising Themes for Linking Intervention with Developmental Science. Ann N Y Acad Sci. 2006;1094(1):125-38.
5. Garland EL, Boettiger CA, Howard MO. Targeting cognitive-affective risk mechanisms in stress-precipitated alcohol dependence: An integrated, biopsychosocial model of automaticity, allostasis, and addiction. Med Hypotheses. 2011;76(5):745-54.
6. Buckner JC, Mezzacappa E, Beardslee WR. Self-Regulation and Its Relations to Adaptive Functioning in Low Income Youths. Am J Orthopsychiatry. 2009;79(1):19-30.
7. Murray DW, K. R, Christopoulos C. An Applied Contextual Model for Promoting Self-Regulation Enactment Across Development: Implications for Prevention, Public Health and Future Research. The Journal of Primary Prevention. 2019;40:367-403.
8. Biripi Aboriginal Corporation Medical Centre. Nyiirunba Yabang (Our path) 2020-2023. Biripi ACMC.; 2020.
9. Australian Commission on Safety and Quality in Health Care. National Safety and Quality Health Service Standards: User Guide for Aboriginal and Torres Strait Islander Health. Sydney: Australian Commission on Safety and Quality in Health Care. . 2017.
10. Prodcutivity Commission. PC Productivity Insights: Australia’s long term productivity experience.; 2020.
11. National Mental Health Commission. National Children’s Mental Health and Wellbeing Strategy 2021. 2021.
12. NSW Health. The First 2000 Days Framework. 2019.
13. Health. AGDo. National action plan for the health of children and young people: 2020–2030. 2019.
14. Woolfenden S, Eapen V, Jalaludin B, Hayen A, Kemp L, Dissanyake C, et al. Prevalence and factors associated with parental concerns about development detected by the Parents’ Evaluation of Developmental Status (PEDS) at 6-month, 12-month and 18-month well-child checks in a birth cohort. BMJ Open. 2016;6(9):e012144.
15. Woolfenden S, Eapen V, Williams K, Hayen A, Spencer N, Kemp L. A systematic review of the prevalence of parental concerns measured by the Parents’ Evaluation of Developmental Status (PEDS) indicating developmental risk. BMC Pediatr. 2014;14(1):231.
16. COAG. COAG Health Council 2019 The Australian National Breastfeeding Strategy: 2019 and Beyond was prepared under the auspices of the COAG Health Council. 2019.
17. Moore T, Arefadib N, Deery A, Keyes M, West S. The first thousand days: an evidence paper. Melbourne: Murdoch Children’s Research Institute. 2017.

18. Goldfeld S, D'Abaco E, Bryson H, Mensah F, Price AM. Surveying social adversity in pregnancy: The antenatal risk burden experienced by Australian women. Journal of paediatrics and child health. 2018;54(7):754-60.
19. Woolfenden S, Galea C, Badland H, Sheedy HS, Williams K, Kavanagh AM, et al. Use of health services by preschool-aged children who are developmentally vulnerable and socioeconomically disadvantaged: testing the inverse care law. J Epidemiol Community Health. 2020;74(6):495-501.
20. Woolfenden S, Galea C, Badland H, Smithers-Sheedy H, Williams K, Kavanagh A, et al. The intersection of developmental vulnerability and socioeconomic disadvantage on access to health care for preschool aged children: evidence for the inverse-care law. Journal of Epidemiology & Community Health(submitted and under review). 2019.
21. Chando S, Craig JC, Burgess L, Sherriff S, Purcell A, Gunasekera H, et al. Developmental risk among Aboriginal children living in urban areas in Australia: the Study of Environment on Aboriginal Resilience and Child Health (SEARCH). BMC pediatrics. 2020;20(1):1-13.
22. Mei C, Nelson B, Hartmann J, Spooner R, McGorry PD. Transdiagnostic early intervention, prevention, and prediction in psychiatry. Personalized Psychiatry: Elsevier; 2020. p. 27-37.
23. Teager W, Fox S, Stafford N. How Australia can invest early and return more: A new look at the $15b cost and opportunity. Australia: Early Intervention Foundation, The Front Project and CoLab at the Telethon Kids Institute; 2019.
24. Baker J, Kohlhoff J, Onobrakpor S-I, Woolfenden S, Smith R, Knebel C, Eapen V. The Acceptability and Effectiveness of Web-Based Developmental Surveillance Programs: Rapid Review. JMIR mHealth and uHealth. 2020;8(4):e16085.
25. Peyton D, Goods M, Hiscock H. The Effect of Digital Health Interventions on Parents' Mental Health Literacy and Help Seeking for Their Child's Mental Health Problem: Systematic Review. Journal of Medical Internet Research. 2022;24(2):e28771.
26. Glover J, et al. The effectiveness of place-based interventions in improving development, health and well-being outcomes in children aged 0-6 years living in disadvantaged neighbourhoods. . Wellbeing, Space and Society (in press) 2021.
27. Cibralic S, Kohlhoff J, Wallace N, McMahon C, Eapen V. A systematic review of emotion regulation in children with Autism Spectrum Disorder. Research in Autism Spectrum Disorders. 2019;68:101422.
28. Noble K, Hurley P, Macklin S. COVID-19, employment stress and student vulnerability in Australia. 2020.
29. Karlov L, Masi A, Diaz AM, Khan F, Winata T, Gilbert M, et al. A Preliminary Trial of an Early Surveillance Program for Autism and Developmental Delays within General Practices. Journal of Developmental and Physical Disabilities. 2024:1-11.
30. Kohlhoff J, Dadich A, Varghese J, McKenzie A, Ong N, Pritchard M, et al. Consumer and health professional perceptions of Watch Me Grow-Electronic (WMG-E) platform for developmental surveillance in early childhood: A qualitative study. Australian Journal of General Practice. 2022;51(6):439-45.
31. Allison C, Baron-Cohen S, Wheelwright S, Charman T, Richler J, Pasco G, Brayne C. The Q-CHAT (Quantitative CHecklist for Autism in Toddlers): a normally distributed quantitative measure of autistic traits at 18–24 months of age: preliminary report. Journal of autism and developmental disorders. 2008;38:1414-25.
32. Sheldrick RC, Perrin EC. Evidence-based milestones for surveillance of cognitive, language, and motor development. Academic pediatrics. 2013;13(6):577-86.

33. Kessler RC, Barker PR, Colpe LJ, Epstein JF, Gfroerer JC, Hiripi E, et al. Screening for Serious Mental Illness in the General Population. Archives of General Psychiatry. 2003;60(2):184-9.
34. Garg A, Butz AM, Dworkin PH, Lewis RA, Thompson RE, Serwint JR. Improving the management of family psychosocial problems at low-income children's well-child care visits: The WE CARE project. Pediatrics 2007;120(3):547-58.
35. Fishman KN, Levitt AJ, Markoulakis R, Weingust S. Satisfaction with mental health navigation services: Piloting an evaluation with a new scale. Community Mental Health Journal. 2018;54:521-32.
36. Kirk MA, Kelley C, Yankey N, Birken SA, Abadie B, Damschroder L. A systematic review of the use of the Consolidated Framework for Implementation Research. Implementation Science. 2015;11:1-13.
37. Proctor E, Silmere H, Raghavan R, Hovmand P, Aarons G, Bunger A, et al. Outcomes for implementation research: conceptual distinctions, measurement challenges, and research agenda. Administration and policy in mental health and mental health services research. 2011;38:65-76.
38. Malterud K, Siersma VD, Guassora AD. Sample size in qualitative interview studies: guided by information power. Qualitative health research. 2016;26(13):1753-60.
39. Braun V, Clarke V. Using thematic analysis in psychology. Qualitative research in psychology. 2006;3(2):77-101.
40. Woolfenden S, Posada N, Krchnakova R, Crawford J, Gilbert J, Jursik B, et al. Equitable access to developmental surveillance and early intervention - understanding the barriers for children from culturally and linguistically diverse (CALD) backgrounds. Health Expect doi: 101111/hex12318. 2014.

10. APPENDIX

Appendix A: Demographic questions

We are going to ask you some questions regarding yourself and your child. This information will help us understand how best to support families like yours and connect you with the services you need.

Please note that all answers will remain confidential. We encourage you to answer the questions, as your responses will assist us in connecting you with the appropriate services. However, some of the questions are optional; if you prefer not to answer you can leave them blank.

- Parent's/Carer's relationship to the child (required)
- Parent's/Carer's year of birth *(categories/range of years)*

1. Child's first name (required)
2. Child's sex (Male / Female)
3. Child's date of birth (required)
4. Child's age (in months) (required)
5. Was your child premature? (Yes / No): If yes, at how many weeks did delivery happen?

- Parent's/Carer's cultural or ethnic background
- *Understanding the rich diversity within our community helps us ensure that our services are inclusive and culturally responsive to the unique needs of all individuals and families.*
- Parent's/Carer's country of birth

6. Do you or your children identify as Aboriginal or Torres Strait Islander? (Yes / No)

- Main language spoken at home

<u>Primary outcome measure - *Developmental surveillance*</u>

1) Have you previously completed the developmental checks in the Blue Book in NSW/ Purple Book in WA? (required) (Yes / No)

2) If yes, at what age did you previously complete the developmental checks? (required) (Tick as many as applicable)
6 months
12 months
18 months
24 months
36 months
48 months
60 months

Appendix B: Learn the Signs Act Early (LTSAE) assessment

Please note, this is an age-specific assessment. A 6 month checklist is attached as an example.

What most babies do by this age:

Social/Emotional Milestones

- ☐ Knows familiar people
- ☐ Likes to look at himself in a mirror
- ☐ Laughs

Language/Communication Milestones

- ☐ Takes turns making sounds with you
- ☐ Blows "raspberries" (sticks tongue out and blows)
- ☐ Makes squealing noises

Cognitive Milestones (learning, thinking, problem-solving)

- ☐ Puts things in her mouth to explore them
- ☐ Reaches to grab a toy he wants
- ☐ Closes lips to show she doesn't want more food

Movement/Physical Development Milestones

- ☐ Rolls from tummy to back
- ☐ Pushes up with straight arms when on tummy
- ☐ Leans on hands to support himself when sitting

Other important things to share with the doctor...

- What are some things you and your baby do together?
- What are some things your baby likes to do?
- Is there anything your baby does or does not do that concerns you?
- Has your baby lost any skills he/she once had?
- Does your baby have any special healthcare needs or was he/she born prematurely?

Appendix C: Quantitative Checklist for Autism in Toddlers (Q-CHAT-10) assessment

For each item, please circle the response which best applies to your child:

		A	B	C	D	E
1	Does your child look at you when you call his/her name?	Always	Usually	Sometimes	Rarely	Never
2	How easy is it for you to get eye contact with your child?	Very easy	Quite easy	Quite difficult	Very difficult	Impossible
3	Does your child point to indicate that s/he wants something? (e.g. a toy that is out of reach)	Many times a day	A few times a day	A few times a week	Less than once a week	Never
4	Does your child point to share interest with you? (e.g. pointing at an interesting sight)	Many times a day	A few times a day	A few times a week	Less than once a week	Never
5	Does your child pretend? (e.g. care for dolls, talk on a toy phone)	Many times a day	A few times a day	A few times a week	Less than once a week	Never
6	Does your child follow where you're looking?	Many times a day	A few times a day	A few times a week	Less than once a week	Never
7	If you or someone else in the family is visibly upset, does your child show signs of wanting to comfort them? (e.g. stroking hair, hugging them)	Always	Usually	Sometimes	Rarely	Never
8	Would you describe your child's first words as:	Very typical	Quite typical	Slightly unusual	Very unusual	My child doesn't speak
9	Does your child use simple gestures? (e.g. wave goodbye)	Many times a day	A few times a day	A few times a week	Less than once a week	Never
10	Does your child stare at nothing with no apparent purpose?	Many times a day	A few times a day	A few times a week	Less than once a week	Never

Appendix D: Survey of Wellbeing of Young Children (SWYC) Baby and Preschool Paediatric checklist

Please note, this is an age-specific assessment. A 6 month checklist is attached as an example.

BABY PEDIATRIC SYMPTOM CHECKLIST (BPSC)			
These questions are about your child's behavior. Think about what you would expect of other children the same age, and tell us how much each statement applies to your child.			
	**Not at all**	**Somewhat**	**Very Much**
Does your child have a hard time being with new people?	0	1	2
Does your child have a hard time in new places?	0	1	2
Does your child have a hard time with change?	0	1	2
Does your child mind being held by other people?	0	1	2
Does your child cry a lot?	0	1	2
Does your child have a hard time calming down?	0	1	2
Is your child fussy or irritable?	0	1	2
Is it hard to comfort your child?	0	1	2
Is it hard to keep your child on a schedule or routine?	0	1	2
Is it hard to put your child to sleep?	0	1	2
Is it hard to get enough sleep because of your child?	0	1	2
Does your child have trouble staying asleep?	0	1	2

Appendix E: Kessler's Psychological Distress Scale (K10)

For all questions, please select the appropriate response.

In the past 4 weeks:	None of the time	A little of the time	Some of the time	Most of the time	All of the time
1. About how often did you feel tired out for no good reason?	☐	☐	☐	☐	☐
2. About how often did you feel nervous?	☐	☐	☐	☐	☐
3. About how often did you feel so nervous that nothing could calm you down?	☐	☐	☐	☐	☐
4. About how often did you feel hopeless?	☐	☐	☐	☐	☐
5 About how often did you feel restless or fidgety?	☐	☐	☐	☐	☐
6. About how often did you feel so restless you could not sit still?	☐	☐	☐	☐	☐
7. About how often did you feel depressed?	☐	☐	☐	☐	☐
8. About how often did you feel that everything was an effort?	☐	☐	☐	☐	☐
9. About how often did you feel so sad that nothing could cheer you up?	☐	☐	☐	☐	☐
10. About how often did you feel worthless?	☐	☐	☐	☐	☐

Appendix F: WE CARE questionnaire

Children and their families thrive when their parents/carers are supported. Our service can help families connect with community resources that provide support. Many of these resources are free of charge. We are asking all families to complete the following survey in the waiting room. We are providing all families with a free information pack about community resources that provide support.

Completing this survey will help us understand what supports families need from our service to thrive.

Child full name: ______________________________ **Child date of birth:** ____________________

Who is completing this survey? D Mother D Father D Other, please specify: ____________________

Do you want help with?

Childcare or schooling
D Yes D No D Prefer not to answer

Government benefits & vouchers
D Yes D No D Prefer not to answer

Housing
D Yes D No D Prefer not to answer

Food
D Yes D No D Prefer not to answer

Bills
D Yes D No D Prefer not to answer

Transport
D Yes D No D Prefer not to answer

Using the NDIS
D Yes D No D Prefer not to answer

Do you need urgent help? (Immediate referral to Kids Rehab Social Worker)

Do you think you are at risk of becoming homeless?

D Yes D No

Is this an emergency, do you need food for tonight?

D Yes D No

Did you feel comfortable answering these questions?

D	D	D	D	D
Very uncomfortable	Somewhat uncomfortable	Neither comfortable nor uncomfortable	Somewhat Comfortable	Very comfortable

You can tell us what made you feel comfortable or uncomfortable below:

____

____

We have given all families a free information pack about community resources that provide support for the above things. Would you also like someone from our team to contact you via phone to discuss any of the things from this survey?

D Yes D No

Appendix G: Semi-structured questionnaire for service use

1. Have you and/or your child attended any of the following services? (required)
   - Psychologist
   - Child and Family health nurse
   - Social worker
   - Paediatrician
   - Occupational therapist
   - Speech pathologist
   - Early childhood education/intervention centres
   - Hubs
   - Other services (please specify) ________

2. If they tick any of the above, display of following questions **for each service:**
   - Who is receiving the service(s)? (me/child/both)
   - When did you and/or child begin attending the service(s)? (state month and year, e.g. July 2020)
   - How frequently do you and/or attend the service(s)? (daily, weekly, fortnightly, monthly, bi-monthly (every 2 months), quarterly, half yearly (every 6 months), annually, other (please specify)).
   - Where did you hear about the service(s)?
   - Was there any service you could not access?
   - How long did you have wait to access the service(s)?

   - How satisfied do you feel with the service(s)?

1	2	3	4	5	6	7	8	9	10
Extremely dissatisfied				Neutral					Extremely satisfied

Appendix H: Questions about Racism

Understanding the rich diversity within our community helps us ensure that our services are inclusive and culturally responsive to the unique needs of all individuals and families. With this in mind, we would like to ask you a couple of questions related to racism. Your responses will help us better understand the experiences of families in our community as well as address any concerns and ensure that our services are accessible and equitable to all.
Please note that your responses are voluntary, and you have the option to skip any question or provide an answer along with someone in our team if needed. Your privacy and comfort are of utmost importance to us.

1. Have you ever been treated unfairly or discriminated against or been treated badly because of your race, ethnicity or cultural background?
2. How often does your family experience racism, discrimination or prejudice?
3. As far as you know, has your child been bullied or treated unfairly at preschool/school because of his/her race, ethnicity or cultural background?
4. If you have experienced racism, discrimination or prejudice, how does this impact your health, access to services, and community connectedness?

	Not at all	Slightly	Moderately	Significantly	Extremely
Health					
Access to services					
Community connectedness					

Appendix I: EuroQoL-5 Dimensions (EQ-5D)

Making any EQ-5D (sample) version available on a publicly accessible webpage is **not allowed**. For reproduction/ displaying any EQ-5D sample version, please submit a request for permission by using the EQ-5D registration form.

Figure 1 / UK (English) EQ-5D-5L Paper Self-Complete (sample version)

Under each heading, please tick the ONE box that best describes your health TODAY.

MOBILITY

I have no problems in walking about ☐
I have slight problems in walking about ☐
I have moderate problems in walking about ☐
I have severe problems in walking about ☐
I am unable to walk about ☐

SELF-CARE

I have no problems washing or dressing myself ☐
I have slight problems washing or dressing myself ☐
I have moderate problems washing or dressing myself ☐
I have severe problems washing or dressing myself ☐
I am unable to wash or dress myself ☐

USUAL ACTIVITIES (*e.g. work, study, housework, family or leisure activities*)

I have no problems doing my usual activities ☐
I have slight problems doing my usual activities ☐
I have moderate problems doing my usual activities ☐
I have severe problems doing my usual activities ☐
I am unable to do my usual activities ☐

PAIN / DISCOMFORT

I have no pain or discomfort ☐
I have slight pain or discomfort ☐
I have moderate pain or discomfort ☐
I have severe pain or discomfort ☐
I have extreme pain or discomfort ☐

ANXIETY / DEPRESSION

I am not anxious or depressed ☐
I am slightly anxious or depressed ☐
I am moderately anxious or depressed ☐
I am severely anxious or depressed ☐
I am extremely anxious or depressed ☐

SAMPLE

© EuroQol Research Foundation. EQ-5D™ is a trade mark of the EuroQol Research Foundation

- We would like to know how good or bad your health is TODAY.
- This scale is numbered from O to 100.
- 100 means the <u>best</u> health you can imagine.
  O means the <u>worst</u> health1you can imag1ine.
- Mark an X on the scale to iindicate how your heallth iis TODAY.
- Now,. please wriite the 11umiber you marked on the scale in the box below.

The best health
you can imagine

100
95
90
85
80
75
70
65
60
55
50
45
40
35
30
25
20
15
10
5
0

The worst health
you can imagine

YOUR HEALiH TODAY= D

Appendix J: <u>The Caregiver Indirect and Informal Care Cost Assessment Questionnaire</u>

We will now ask you questions regarding your current work status and the time that you spend caring for your child.

<u>WORK STATUS</u>

1) Are you currently employed or self-employed (working for pay)?
Yes/No

2) How many hours per week do you work (e.g., according to your employment contract)?

3) Are you currently working full-time?
Yes/No. If Yes, skip to question 6.

4) Did you reduce your working hours due to your child? (e.g., to care for him/her)?
Yes/No

5) How many hours per week did you work before reducing your working hours?

6) During the <u>last week</u>, how many hours did you miss from work due to your child?

7) During the <u>last week</u>, how much did your child affect your productivity while you were working?
0-10 (from could work as usual to could not work at all) – Go to Q9

8) Did you stop working because of your child? Yes/No

9) Are you compensated in any way for the time that you care for your child?
Yes/No

<u>INFORMAL CAREGIVING</u>

10) During the last week, how much time did you spend on household activities and tasks looking after your child?

11) During the last week, how much time did you spend helping your child with her/his personal care?

12) During the last week, how much time did you spend providing practical support to your child?

13) During the last week, how much time did you spend providing emotional support to your child?

Appendix K: Patient Satisfaction Questionnaire

	Disagree	Tend to disagree	Neither agree or disagree	Tend to agree	Agree
1. I am better able to deal with issues that I sought help with					
2. I have developed meaningful connections and friendships within the hub community					
3. I feel more connected to available social and health services					
4. I am satisfied with the services I have received					
5. The service listened to me and understood my issues					
6. Any other feedback about the program or staff?					

Appendix L: NAVSAT questionnaire

Part I: Navigation Service

Item	1	2	3	4	5	6	7
1. How satisfied are you with the Navigator's ability to listen and understand your concerns?	Extremely Dissatisfied	Dissatisfied	Fairly Dissatisfied	Not dissatisfied nor satisfied	Fairly Satisfied	Satisfied	Extremely Satisfied
2. How satisfied are you with the information given about potential treatment options for your child/family member?	Extremely Dissatisfied	Dissatisfied	Fairly Dissatisfied	Not dissatisfied nor satisfied	Fairly Satisfied	Satisfied	Extremely Satisfied
3. How satisfied are you with how the Navigator understood the impact of the situation on your family?	Extremely Dissatisfied	Dissatisfied	Fairly Dissatisfied	Not dissatisfied nor satisfied	Fairly Satisfied	Satisfied	Extremely Satisfied
4. How satisfied are you with the Navigator's confidentiality and respect for you and your child/family member's rights?	Extremely Dissatisfied	Dissatisfied	Fairly Dissatisfied	Not dissatisfied nor satisfied	Fairly Satisfied	Satisfied	Extremely Satisfied
5. How satisfied are you with the intake procedures?	Extremely Dissatisfied	Dissatisfied	Fairly Dissatisfied	Not dissatisfied nor satisfied	Fairly Satisfied	Satisfied	Extremely Satisfied
6. How satisfied are you with your frequency of contact with the Navigator?	Extremely Dissatisfied	Dissatisfied	Fairly Dissatisfied	Not dissatisfied nor satisfied	Fairly Satisfied	Satisfied	Extremely Satisfied
7. How frequently did you have contact with the Navigator?	Less than once every 2 months	Once every 1-2 months	Once every month	Once every three weeks	Once every two weeks	Once per week	More than once per week
8. To what degree do you feel the Navigator recommended the most appropriate resources for your child/family member?	Extremely Small Degree	Small Degree	Fairly Small Degree	Moderate Degree	Fairly Large Degree	Large Degree	Extremely Large Degree
9. To what degree do you feel the Navigator truly understands the mental health system?	Extremely Small Degree	Small Degree	Fairly Small Degree	Moderate Degree	Fairly Large Degree	Large Degree	Extremely Large Degree
10. In general, how helpful did you find the Navigator?	Extremely Unhelpful	Unhelpful	Fairly Unhelpful	Not helpful nor unhelpful	Fairly Helpful	Helpful	Extremely Helpful
11. How likely are you to recommend this service to family and friends?	Very Unlikely	Unlikely		Not Sure	Likely		Very Likely
12. In general, how satisfied are you with the Family Navigation Project?	Extremely Dissatisfied	Dissatisfied	Fairly Dissatisfied	Not dissatisfied nor satisfied	Fairly Satisfied	Satisfied	Extremely Satisfied

Part II: Referred Service

Item	1	2	3	4	5	6	7
4. How effective has the referred service been in giving you advice to deal better with your child/family member's issues?	Extremely Ineffective	Ineffective	Fairly Ineffective	Neither Effective nor Ineffective	Fairly Effective	Effective	Extremely Effective
5. How effective has the referred service been in improving your child/family member's wellbeing?	Extremely Ineffective	Ineffective	Fairly Ineffective	Neither Effective nor Ineffective	Fairly Effective	Effective	Extremely Effective
6. How satisfied are you with the information given to you by the referred service about the youth/family member's treatment procedure?	Extremely Dissatisfied	Dissatisfied	Fairly Dissatisfied	Not dissatisfied nor satisfied	Fairly Satisfied	Satisfied	Extremely Satisfied
7. How satisfied are you with the communication you had/have with the clinician/team of the referred service?	Extremely Dissatisfied	Dissatisfied	Fairly Dissatisfied	Not dissatisfied nor satisfied	Fairly Satisfied	Satisfied	Extremely Satisfied
8. How satisfied are you with the referred service's ability to listen and understand your child/family member?	Extremely Dissatisfied	Dissatisfied	Fairly Dissatisfied	Not dissatisfied nor satisfied	Fairly Satisfied	Satisfied	Extremely Satisfied
9. How satisfied are you with the frequency of your contact/dialogue with the referred service?	Extremely Dissatisfied	Dissatisfied	Fairly Dissatisfied	Not dissatisfied nor satisfied	Fairly Satisfied	Satisfied	Extremely Satisfied
10. In general, how satisfied are you with the referred service?	Extremely Dissatisfied	Dissatisfied	Fairly Dissatisfied	Not dissatisfied nor satisfied	Fairly Satisfied	Satisfied	Extremely Satisfied

Appendix M: Brief Semi-structured questions for control group at 6 and 18 mid-points.

Part 1: *Child Development*

1. Have you completed your baby's developmental checks in the NSW Blue Book or WA Purple Book? (required) (Yes / No)

2. (If no to Q1), what was the reason you couldn't complete the development checks? (required)

- Didn't know where to go
- Didn't have the time
- Didn't have transport to travel to checks
- Didn't feel it was necessary
- Other reason

(If other reason selected) – Please explain the barrier you faced? free text box

3. (If yes to Q1) Which checks have you completed to date? (required) (Tick all that apply)

- 1-4 weeks
- 6-8 weeks
- 6 months
- 12 months
- 18 months
- 24 months
- 36 months

4. (If yes to Q1) Who did you complete the last check with? (required)

- General Practitioner (GP)
- CFH Nurse (at hospital/Hub/CFH Centre)
- GP Nurse
- NSW Refugee Health
- Other – Please specify

5. (If yes to Q1) Did your child have any concerns identified while doing the checks? (required)

- Yes
- No

6. What was the concern?

- Gross motor *(This is about how your child uses their large muscles to move around)* For example: crawling, walking, jumping, or climbing.
- Fine motor (*This is about how your child uses their hands and fingers to do things)* For example: picking up small objects, feeding themselves, or drawing.
- Communication *(This is how your child understands and uses language)* For example: making sounds, pointing, using words, or following directions.
- Cognitive *(This is how your child learns, thinks, and solves problems)* For example: exploring toys, figuring out puzzles, or remembering routines.

- Social emotional *(This is how your child interacts with others and expresses emotions)* For example: smiling, playing with others, showing affection, or managing feelings.

7a. (If yes to Q1) Did your child receive any referrals or support? (required)
- Yes
- No

7b. (If yes to Q1) Were you able to access the referred services? (required)
- Yes
- No

8. (If yes to Q1 AND If yes to Q8a) what referrals or support services did your child receive? (required)
  - o Psychologist
  - o Child and Family health nurse
  - o Social care services
  - o Paediatrician
  - o Allied health services (Occupational therapist, speech pathologist, physiotherapist)
  - o Medical or surgical specialists (not including GP)
  - o Early childhood education/intervention centres
  - o Hubs
  - o Other services (please specify) _______
  - o None of the above

9. (If yes to Q1 AND If yes to Q8a) How satisfied do you feel with the service(s)?

☹ ☺

1 2 3 4 5 6 7 8 9 10
Extremely dissatisfied Neutral Extremely satisfied

10. What would have improved your experience? Free text box

11. (If yes to Q1 and no to Q8b) Please let us know why you couldn't use any of the services. (required)
- The support I needed was different to what was offered
- I tried, but waiting time/process was too long
- Relevant services were not available in the area where I live
- Other (language or cultural issues), please explain

(If option 1 selected in Q8b) Please explain: ____________________

Part 2: *<u>Parent Mental Health</u>*

1. In the past 6 months, have you received referrals for support services for your mental health? (required)
- Yes
- No

2. (If yes to Q1) Were you able to access these support services? (required)
- Yes
- No

3. (If yes to Q2) what services have you been referred to or received? (Tick all that apply) (required)
- Psychologist
- Allied health (speech, OT, physiotherapy)
- General Practitioner
- Social care services (example: housing, financial counselling)
- NDIS
- Medical or surgical specialists (not including GP)
- Hubs
- Other services (please specify) ________
- None of the above

4. (If yes to Q2) How satisfied do you feel with the service(s)?

☹ ☺

1 2 3 4 5 6 7 8 9 10

Extremely dissatisfied Neutral Extremely satisfied

5. What would have improved your experience? Free text box

6. (if yes to Q1 and no to Q2) Please let us know why you couldn't use any of the services. (required)
- The support I needed was different to what was offered
- I tried, but waiting time/process was too long
- Relevant services were not available in the area where I live
- Other, please explain

7. (If option 1 selected in Q6) Please explain: ______________________

Part 3: *Family psychosocial needs*

1. In the past 6 months, did you need support with any social care needs? (required)
   - Yes
   - No

2. (If yes to Q1) Were you able to access the support you needed? (required)
   - Yes
   - No

3. (If yes to Q2), what did you need support with? (please select all that apply) (required)
   - Childcare or schooling
   - Government benefits and vouchers
   - Housing
   - Food
   - Bills
   - Transport
   - Other, please specify:

4. (If yes to Q2) How satisfied do you feel with the service(s)?

☹ ☺

1 2 3 4 5 6 7 8 9 10

Extremely dissatisfied Neutral Extremely satisfied

5. What would have improved your experience? Free text box

6. (If yes to Q1 AND if no to Q2) Please let us know why you couldn’t use any of the services. (required)
   - The support I needed was different to what was offered
   - I tried, but waiting time/process was too long
   - Relevant services were not available in the area where I live
   - Other, please explain

7. (If option 1 selected in Q6) Please explain: ______________________

Appendix N: Patient-Reported Outcomes Measurement Information System Early Childhood Parent Report (PROMIS EC) Childhood Wellbeing (Self-regulation subscale)

Q1 Please think about your child's behaviour in the **past week** when selecting your responses. Please respond to each question or statement by marking one box per row.

Please respond to each question or statement by marking one box per row.

In the past 7 days

		Never	Rarely	Sometimes	Often	Always
selfreg_ec1	My child made transitions easily	☐ 1	☐ 2	☐ 3	☐ 4	☐ 5
selfreg_ec2	My child accepted suggestions for alternative ways of doing something	☐ 1	☐ 2	☐ 3	☐ 4	☐ 5
selfreg_ec3	My child was flexible to change	☐ 1	☐ 2	☐ 3	☐ 4	☐ 5
selfreg_ec4	My child adapted well to setbacks or challenges..	☐ 1	☐ 2	☐ 3	☐ 4	☐ 5
selfreg_ec5	My child adjusted easily to changes in routines..	☐ 1	☐ 2	☐ 3	☐ 4	☐ 5

In the past 7 days

		Never	Rarely	Sometimes	Often	Always
selfreg_ec6	My child could wait if asked, even if he/she really wanted to do something.......	☐ 1	☐ 2	☐ 3	☐ 4	☐ 5
selfreg_ec7	My child managed frustration well...........	☐ 1	☐ 2	☐ 3	☐ 4	☐ 5
selfreg_ec8	My child stayed calm and in control when faced with a challenge...............................	☐ 1	☐ 2	☐ 3	☐ 4	☐ 5
selfreg_ec11	My child found ways to distract him/herself when frustrated	☐ 1	☐ 2	☐ 3	☐ 4	☐ 5
selfreg_ec14	My child bounced back quickly when things didn't go his/her way......................	☐ 1	☐ 2	☐ 3	☐ 4	☐ 5
selfreg_ec15r1	My child was good at managing his/her behaviors	☐ 1	☐ 2	☐ 3	☐ 4	☐ 5

Appendix O: Multidimensional Assessment Profile of Disruptive Behavior Infant-Toddler (MAP-DB-IT) Temper Loss

Q11.1 We are interested in learning about your child’s behaviour and feelings when angry, upset or frustrated. Please answer the following questions about **the past month** with your child .You may notice that more than one question asks about similar behaviors or feelings. This is because children may express the same thing in different ways. Thank you for responding to each item to provide us with a detailed picture of how your child manages his/her behavior and feelings.

	Never in past month	Rarely (less than once per week)	Some (1-3) days of the week	Most (4-6) days of the week	Every day	Many times each day
1. Hit, bite, or kick during a temper tantrum						
2. Hold breath during a temper tantrum						
3. Have a temper tantrum until s/he was exhausted						
4. Break or destroy things during a temper tantrum						
5. React explosively when mad						
6. Stamp his/her feet when frustrated						
7. Have a temper tantrum that lasted longer than 5â€‰minutes						
8. Keep on having a temper tantrum even when you tried to help him/her calm down						
9. Fall to the floor when frustrated						
10. Scream loudly when frustrated						
11. Cry when frustrated						
12. Have a temper tantrum						
13. Have a temper tantrum when s/he was with other adults (e.g., babysitter, childcare provider, teacher, family member)						
14. Have a temper tantrum when s/he was with you or other parent						
15. Suddenly have a temper tantrum that came out of nowhere						
16. Have a temper tantrum when you were out in public with him/her						
17. Have a temper tantrum when asked to stop playing and do something else						
18. Have a temper tantrum during daily routines such as bedtime, mealtime, or getting dressed						
19. Become frustrated about small things						

20. Have a temper tantrum when s/he was tired, hungry, or sick						
21. Have a temper tantrum when s/he was frustrated, angry, or upset						
22. Stay angry for a long time						
23. Act grumpy during fun activities						
24. Do things that could hurt him/herself during a temper tantrum (for example, bang head, bite or scratch self)						
25. Wake up grumpy						
26. Get really mad						
27. Have trouble cheering up when grumpy						
28. Fuss for a long time no matter what you do						
29. Act grumpy						
30. Get in an angry mood						

Appendix P: Tool to Measure Parenting Self-Efficacy (TOPSE)

Q12.1 We will now ask you a few questions to understand how confident you feel about parenting.

The following section is about emotion and affection.
Using the scale below, please enter in the boxes how much you agree with each statement.
The scale ranges from 0 (completely disagree) to 10 (completely agree).
You may use any number between 0 and 10. Please answer all statements.

0	1	2	3	4	5	6	7	8	9	10
Completely disagree				Moderately agree				Completely agree		

- I am able to show affection towards my child.
- I can recognise when my child is happy or sad.
- I am confident my child can come to me if they're unhappy.
- When my child is sad I understand why.
- I have a good relationship with my child.
- I find it hard to cuddle my child.

The following section is about empathy and understanding.
Using the scale below, please enter in the boxes how much you agree with each statement.
The scale ranges from 0 (completely disagree) to 10 (completely agree).
You may use any number between 0 and 10. Please answer all statements.

0	1	2	3	4	5	6	7	8	9	10
Completely disagree				Moderately agree				Completely agree		

- I am able to explain things patiently to my child.
- I can get my child to listen to me.
- I am able to comfort my child.
- I am able to listen to my child.
- I am able to put myself in my child's shoes.
- I understand my child's needs.

The following section is about play and enjoyment.
Using the scale below, please enter in the boxes how much you agree with each statement.
The scale ranges from 0 (completely disagree) to 10 (completely agree).
You may use any number between 0 and 10. Please answer all statements.

0	1	2	3	4	5	6	7	8	9	10
Completely disagree				Moderately agree				Completely agree		

- I am able to have fun with my child.
- I am able to enjoy each stage of my child's development.
- I am able to have nice days with my child.
- I can plan activities that my child will enjoy.
- Playing with my child comes easily to me.
- I am able to help my child reach their full potential.

Appendix Q: **<u>Coping with Toddlers Negative Emotions Scale (CTNES)</u>**

For the following items, please rate the likelihood that you would respond in the ways listed for each item. Please read each item carefully and respond as honestly and sincerely as you can. For each question, please fill in a circle for each item.

**Please rate the likelihood for each item:**	**Very unlikely**			**Medium**			**Very likely**
**1. If my child becomes angry because he wants to play outside and cannot do so for whatever reason (i.e., he is sick, the outside area is unsafe, it is raining), I would:**							
a. Feel upset myself	○	○	○	○	○	○	○
b. Tell my child we will not get to do something else fun (i.e., watch t.v., play games) unless he stops behaving like that	○	○	○	○	○	○	○
c. Tell my child it's ok to be angry	○	○	○	○	○	○	○
d. Soothe my child	○	○	○	○	○	○	○
e. Help my child find something he wants to do inside.	○	○	○	○	○	○	○
f. Tell my child that he is making a big deal out of nothing	○	○	○	○	○	○	○
**2. If my toddler spilled something and made a big mess that was difficult to clean, and then gets upset and cries, I would:**							
a. Comfort my child	○	○	○	○	○	○	○
b. Tell my child that he is overreacting or making a big deal out of nothing	○	○	○	○	○	○	○
c. Remain calm and not let myself get upset by my child's cries	○	○	○	○	○	○	○
d. Punish my child for making a mess, e.g., send him to another room or take something fun away	○	○	○	○	○	○	○
e. Help my child find a way to clean up the mess	○	○	○	○	○	○	○
f. Tell my child that it is ok to be upset	○	○	○	○	○	○	○
**3. If my child loses some prized possession (for example, his favourite blanket or stuffed animal) and reacts with tears, I would:**							
a. Help my child think of other places to look for the toy	○	○	○	○	○	○	○
b. Distract my child, e.g., by using another toy, to make him feel better	○	○	○	○	○	○	○
c. Tell my child that it is not that important	○	○	○	○	○	○	○

d. Tell my child it is his fault for not being careful with the toy	○▪	○▪	○▪	○▪	○▪	○▪	○▪
e. Feel upset myself	○▪	○▪	○▪	○▪	○▪	○▪	○▪
f. Tell my child it is okay to feel sad about the loss	○▪	○▪	○▪	○▪	○▪	○▪	○▪
**4. If my child is afraid of going to the doctor or of getting shots and becomes quite shaky and teary, I would:**							
a. Tell him to shape up or he won't be allowed to do something he likes to do (i.e., go to playground)	○▪	○▪	○▪	○▪	○▪	○▪	○▪
b. Tell my child that it is ok to be nervous or afraid	○▪	○▪	○▪	○▪	○▪	○▪	○▪
c. Tell my child that it's really no big deal	○▪	○▪	○▪	○▪	○▪	○▪	○▪
d. Comfort my child before and/or after the shot	○▪	○▪	○▪	○▪	○▪	○▪	○▪
e. Help him think of ways to make it less scary, like squeezing my hand when he gets a shot	○▪	○▪	○▪	○▪	○▪	○▪	○▪
f. Get nervous myself	○▪	○▪	○▪	○▪	○▪	○▪	○▪
**5. If my child is going to spend the afternoon with an unfamiliar person, e.g., a new babysitter, or at a childcare centre, and becomes nervous and upset because I am leaving him, I would:**							
a. Distract my child, e.g., by playing and talking about all of the fun he will have with the sitter	○▪	○▪	○▪	○▪	○▪	○▪	○▪
b. Feel upset or nervous myself because of my child's reactions	○▪	○▪	○▪	○▪	○▪	○▪	○▪
c. Tell my child that he won't get to do something else enjoyable (i.e., go to playground, get a special snack) if he doesn't stop behaving like that	○▪	○▪	○▪	○▪	○▪	○▪	○▪
d. Tell him that it's nothing to get upset about	○▪	○▪	○▪	○▪	○▪	○▪	○▪
e. Help my child think of things to do that will make it less stressful	○▪	○▪	○▪	○▪	○▪	○▪	○▪
f. Tell my child that it's ok to be upset	○▪	○▪	○▪	○▪	○▪	○▪	○▪
**7. If my child becomes angry because he is not allowed to have a snack (i.e., candy, ice cream) when he wants it, I would:**							

a. Tell my child that he will not get a treat later if he doesn't stop acting like that	○▪	○▪	○▪	○▪	○▪	○▪	○▪
b. Distract child by playing with other toys or games	○▪	○▪	○▪	○▪	○▪	○▪	○▪
c. Tell him that there is no reason to be upset	○▪	○▪	○▪	○▪	○▪	○▪	○▪
d. Tell my child it's okay to feel angry	○▪	○▪	○▪	○▪	○▪	○▪	○▪
e. Help my child think of something to eat that he is allowed to have between meals	○▪	○▪	○▪	○▪	○▪	○▪	○▪
f. Feel angry	○▪	○▪	○▪	○▪	○▪	○▪	○▪
**9. If my child wants me to play with him and I cannot do so right then (i.e., I am on the phone, in the middle of a conversation with someone), and my child becomes upset, I would:**							
a. Feel upset myself	○▪	○▪	○▪	○▪	○▪	○▪	○▪
b. Tell my child that there is nothing to be upset about	○▪	○▪	○▪	○▪	○▪	○▪	○▪
c. Help my child find something to do while he waits for me to play with him.	○▪	○▪	○▪	○▪	○▪	○▪	○▪
d. Tell my child I won't play with him later if he doesn't stop behaving like that	○▪	○▪	○▪	○▪	○▪	○▪	○▪
e. Tell my child it's okay to be upset	○▪	○▪	○▪	○▪	○▪	○▪	○▪
f. Soothe my child to make him feel better	○▪	○▪	○▪	○▪	○▪	○▪	○▪
**10. If my child is playing with a puzzle or shape sorter toy and cannot fit a piece correctly, and gets upset and cries, I would:**							
a. Remain calm and not let myself get upset from his crying	○▪	○▪	○▪	○▪	○▪	○▪	○▪
b. Take the toy away from my child	○▪	○▪	○▪	○▪	○▪	○▪	○▪
c. Comfort my child (e.g., with a pat or a kiss)	○▪	○▪	○▪	○▪	○▪	○▪	○▪
d. Tell my child it's okay to get frustrated and upset	○▪	○▪	○▪	○▪	○▪	○▪	○▪
e. Help my child figure out how to put the piece in correctly	○▪	○▪	○▪	○▪	○▪	○▪	○▪
f. Tell my child it's nothing to cry about	○▪	○▪	○▪	○▪	○▪	○▪	○▪
**11. If my child has climbed onto a piece of playground equipment and gets stuck, and becomes nervous and begins to cry, I would:**							
a. Become anxious myself	○▪	○▪	○▪	○▪	○▪	○▪	○▪

b. Help my child figure out how to get down from the climber	○	○	○	○	○	○	○
c. Tell my child he shouldn't have gone up by himself	○	○	○	○	○	○	○
d. Tell my child its nothing to get upset about	○	○	○	○	○	○	○
e. Comfort my child (e.g., with words or a pat)	○	○	○	○	○	○	○
f. Tell my child it's okay to be afraid	○	○	○	○	○	○	○
