# Supplementary material for: Effect of strengths-based, tiered, accessible resources and supports (STARS) for Kids project, a codesigned hybrid service delivery framework to identify and support child development, parent mental health and psychosocial needs among parents and carers of children aged 6 months to 3 years: a randomised controlled trial protocol
Source: BMJ Open. 2026 Sep 29;16(9):e122902. doi: 10.1136/bmjopen-2026-122902 (PMC13630006; doi:10.1136/bmjopen-2026-122902)
Supplement: Supplementary file 2 [file bmjopen-2026-122902.reviewer_comments.pdf]

PEER REVIEW HISTORY

BMJ Open publishes all reviews undertaken for accepted manuscripts. Reviewers are asked to complete a checklist review form and are provided with free text boxes to elaborate on their assessment. These free text comments are reproduced below.

ARTICLE DETAILS

Title (Provisional)

Effect of Strengths-based, Tiered, Accessible, Resources and Supports (STARS) for Kids project, a codesigned hybrid service delivery framework to identify and support child development, parent mental health, and psychosocial needs among parents and carers of children aged six months to three years: a randomised controlled trial protocol.

Authors

John, James Rufus; Kohlhoff, Jane; Lambiase, Sini; Nicholls, Sophie; Diez, Maite; Doyle, Kerrie E; Barr, Karlen; Boydell, Katherine; Brennan, Clare; Carson, Toni; Lam-Cassettari, Christa; Cole, Jo; Dadich, Ann; Descallar, Joseph; Finlay-Jones, Amy; Foster, Melissa; Goldfeld, Sharon; Grace, Rebekah; Hodgins, Michael; Honisett, Suzy; Katsamangos, Bree; Katz, Ilan; Kemp, Lynn; Khan, Feroza; Kliendienst, Luana; Lawson, Kenny; Lingam, Raghu; McClean, Tom; Morton, Susan M. B.; O'Loughlin, Grainne; Ostojic, Katarina; Page, Andrew; Prescott, Susan; Raman, Shanti; Schmied, Virginia; Walker, Adam K; Wallace, Amy; Young, Rebecca; Silva, Desiree; Downs, Jenny; Woolfenden, Susan; Eapen, Valsamma; Community Consultation Group, Ngarra Wakulda Aboriginal; Group, STARS for Kids Group

---

VERSION 1 - REVIEW

**Reviewer**	**1**
**Name**	**Rizvi, Arjumand**
**Affiliation**	**Aga Khan Medical University, Pediatrics and Child Health**
**Date**	**10-Aug-2026**
**COI**	**None**

1.The abstract omits the nested MTAM-EVP trial, but the full protocol body describes it adequately.

2-The limitations section could be strengthened by addressing additional methodological considerations, including reliance on parent/carer self-report for the primary outcome, potential differential engagement and contamination between study arms, and the risk of attrition in priority populations. The authors should also acknowledge the potential limited

power of the nested RCT and explain how these issues may affect interpretation of the findings.

VERSION 1 - AUTHOR RESPONSE

Reviewer 1

1) The abstract omits the nested MTAM-EVP trial, but the full protocol body describes it adequately.

Author response: *We thank the reviewer for this feedback. We have now added a sentence about the nested MTAM-EVP trial in the abstract section.*

Modifications: *Additionally, a nested trial will evaluate the My Toddler and Me Enhanced Video Program (MTAM-EVP) intervention against enhanced usual care among children identified with emotional dysregulation.*

2) The limitations section could be strengthened by addressing additional methodological considerations, including reliance on parent/carer self-report for the primary outcome, potential differential engagement and contamination between study arms, and the risk of attrition in priority populations. The authors should also acknowledge the potential limited power of the nested RCT and explain how these issues may affect interpretation of the findings.

Author response: *We have now added the methodological considerations to the limitations section. We have been advised by the editor that the strengths and limitations section should contain up to five short bullet points, no longer than one sentence each, that relate specifically to the methods.*

Modifications:

- *The study may be limited by parent/carer self-report of the primary outcome, differential engagement and contamination between study arms, attrition among priority populations, and limited power in the nested trial.*
